# Stepwise evolution and clonal enrichment of gepotidacin resistance in *Neisseria gonorrhoeae*

**DOI:** 10.1128/aac.01706-25

**Published:** 2026-03-11

**Authors:** Linxin Yao, Tingli Tian, Danyang Zou, Xinying Lu, Nanyan Jiang, Xin Feng, Tong Zheng, Zhuojun Tang, Yi Lin, Zhen Ning, Jianping Jiang, Meiping Ye, Pingyu Zhou

**Affiliations:** 1 STD Institute, Shanghai Skin Disease Hospital, Tongji University School of Medicine 481875, Shanghai, China; 2 Department of Dermatology, Xinhua Hospital, Shanghai Jiao Tong University School of Medicine 56694, Shanghai, China; 3 Division of Tuberculosis and HIV/AIDS Prevention, Shanghai Municipal Center for Disease Control and Prevention 117753, Shanghai, China; 4 Institute of Antibiotics, Huashan Hospital, Fudan University 12478, Shanghai, China; Columbia University Irving Medical Center, New York, New York, USA

**Keywords:** *Neisseria gonorrhoeae*, gepotidacin, antimicrobial resistance, stepwise evolution, ParC D86N

## Abstract

Although gepotidacin is a promising oral candidate for treating multidrug-resistant *Neisseria gonorrhoeae*, its resistance mechanisms and clinical implications remain poorly understood. In this study, we collected 989 clinical *N. gonorrhoeae* isolates from 33 hospitals in Shanghai, China (2022–2024). Antimicrobial susceptibility testing showed that gepotidacin exhibited high *in vitro* activity with MIC_50_ and MIC_90_ of 0.5 and 1 μg/mL, respectively. Further investigation identified that elevated gepotidacin MICs were significantly associated with substitutions at GyrA position 92/95 and ParC position 86/87, including GyrA A92P (odds ratio [OR], 4.25; 95% confidence interval [CI], 2.61–6.94) and D95Y (OR, 4.61; 95% CI, 2.82–7.54), as well as ParC D86N (OR, 3.12; 95% CI, 1.99–4.90) and S87N (OR, 5.92; 95% CI, 3.63–9.64). Substitution combinations analyses revealed that GyrA D95A plus ParC D86N, GyrA A92P/D95Y plus ParC S87N, GyrA D95A plus ParC S87N, and GyrA D95G plus ParC D86N were significantly associated with elevated gepotidacin MICs. Notably, these high-risk substitutions were enriched in international clones ST7363 and ST8123. *In vitro* induction experiments demonstrated a stepwise resistance trajectory: from initial diverse QRDR mutations to an intermediate state with GyrA A92P/D95Y, followed by ParC D86N and GyrA A92T/D95A, culminating in high-level resistance, with MICs all reaching 64 μg/mL. We also found that the strain with preexisting GyrA D95A and ParC D86N more readily acquired A92T and developed high-level resistance under gepotidacin exposure. Our study highlights key mutational patterns and clonal backgrounds that promote gepotidacin resistance, emphasizing the need for optimized dosing strategies as well as targeted molecular surveillance to preserve its effectiveness.

## INTRODUCTION

Gonorrhea, caused by *Neisseria gonorrhoeae*, remains a major global public health threat. If left untreated, it can lead to severe complications, such as pelvic inflammatory disease, infertility, ectopic pregnancy, and increased risk of HIV transmission (1). The global burden continues to rise, with an estimated 82 million new infections reported in 2020 alone (2). Countries including the United States, the United Kingdom, and China have reported increasing case numbers in recent years, with incidence reaching historic highs (3–5).

*N. gonorrhoeae* has developed resistance to nearly all antibiotics used for treatment (6, 7). Ceftriaxone remains the last reliably effective option for empiric monotherapy, yet resistance is spreading globally (8–11). *N. gonorrhoeae* strains resistant to third-generation cephalosporins and fluoroquinolones were categorized as high priority by the WHO in 2024 (12). Gepotidacin, a novel triazaacenaphthylene antibiotic, simultaneously targets DNA gyrase (GyrA) and topoisomerase IV (ParC), disrupting bacterial replication through dual enzymatic inhibition (13). This unique mechanism offers an advantage over traditional fluoroquinolones and has demonstrated potent activity against multidrug-resistant *N. gonorrhoeae* strains (13, 14). Phase II and III clinical trials demonstrated high success rates (15, 16). In 2025, the U.S. Food and Drug Administration approved gepotidacin for the treatment of uncomplicated urinary tract infections (uUTIs) caused by susceptible bacteria in female adult and pediatric patients aged 12 years and older who weigh at least 40 kg (17). However, resistance-associated treatment failures have already been reported (18). Subsequent studies identified the ParC D86N substitution as associated with reduced gepotidacin susceptibility, and GyrA A92T was linked to high-level resistance, raising early concerns regarding resistance development and the potential compromise of gepotidacin’s clinical utility (18).

Despite these concerns, the magnitude and scope of gepotidacin resistance remain poorly defined. Large-scale, real-world susceptibility data are limited, and the prevalence of quinolone resistance-determining region (QRDR) mutations relevant to gepotidacin is not well characterized. Furthermore, the genetic and evolutionary trajectories driving resistance under drug pressure have not been systematically investigated. Addressing these gaps is essential to inform clinical deployment of gepotidacin and prevent the emergence and spread of resistance.

To fill these gaps, we conducted a large-scale genomic and phenotypic analysis of 989 *N. gonorrhoeae* clinical isolates collected from 33 hospitals in Shanghai, China, during 2022–2024. We also performed *in vitro* induction experiments to reconstruct stepwise resistance pathway under gepotidacin selection. These findings support the need for optimized dosing strategies and targeted genomic surveillance to preserve gepotidacin’s clinical utility in the face of evolving resistance.

## RESULTS

### Gepotidacin exhibits high *in vitro* activity against clinical *N. gonorrhoeae* isolates

Between January 2022 and July 2024, a total of 989 *N. gonorrhoeae* isolates (one per patient) were collected from 33 hospitals across Shanghai, China (Fig. S1). Most patients were male (89.3%, 883/989), aged over 20 years (93.5%, 925/989), and presented with urethral infections (89.7%, 887/989). A majority of isolates (72.0%, 712/989) were collected from hospitals in suburban districts (Table 1).

**TABLE 1:** Clinical characteristics of 989 *N. gonorrhoea*e isolates

Characteristic	No. (%) of *N. gonorrhoeae* isolates		Total	*P* value
	MIC ≤ MIC_90_ (*n* = 903)	MIC > MIC_90_ (*n* = 86)		
Gender				0.231
Male	810 (89.7)	73 (84.9)	883	
Female	93 (10.3)	13 (15.1)	106	
Age				0.443
Median (IQR)	34 (26–46)	36 (28–51)	989	
0–19	60 (6.6)	4 (4.7)	64	
20–29	271 (30.0)	22 (25.6)	293	
30–39	246 (27.2)	21 (24.4)	267	
40–49	146 (16.2)	15 (17.4)	161	
≥50	180 (19.9)	24 (27.9)	204	
Isolation site				0.239
Cervix	59 (6.5)	8 (9.3)	67	
Urine	3 (0.3)	0 (0.0)	3	
Urethra	811 (89.8)	73 (84.9)	884	
Vagina	30 (3.3)	5 (5.8)	35	
Geographical distribution				0.917
Urban	252 (27.9)	25 (29.1)	277	
Suburban	651 (72.1)	61 (70.9)	712	

**Fig 1:**
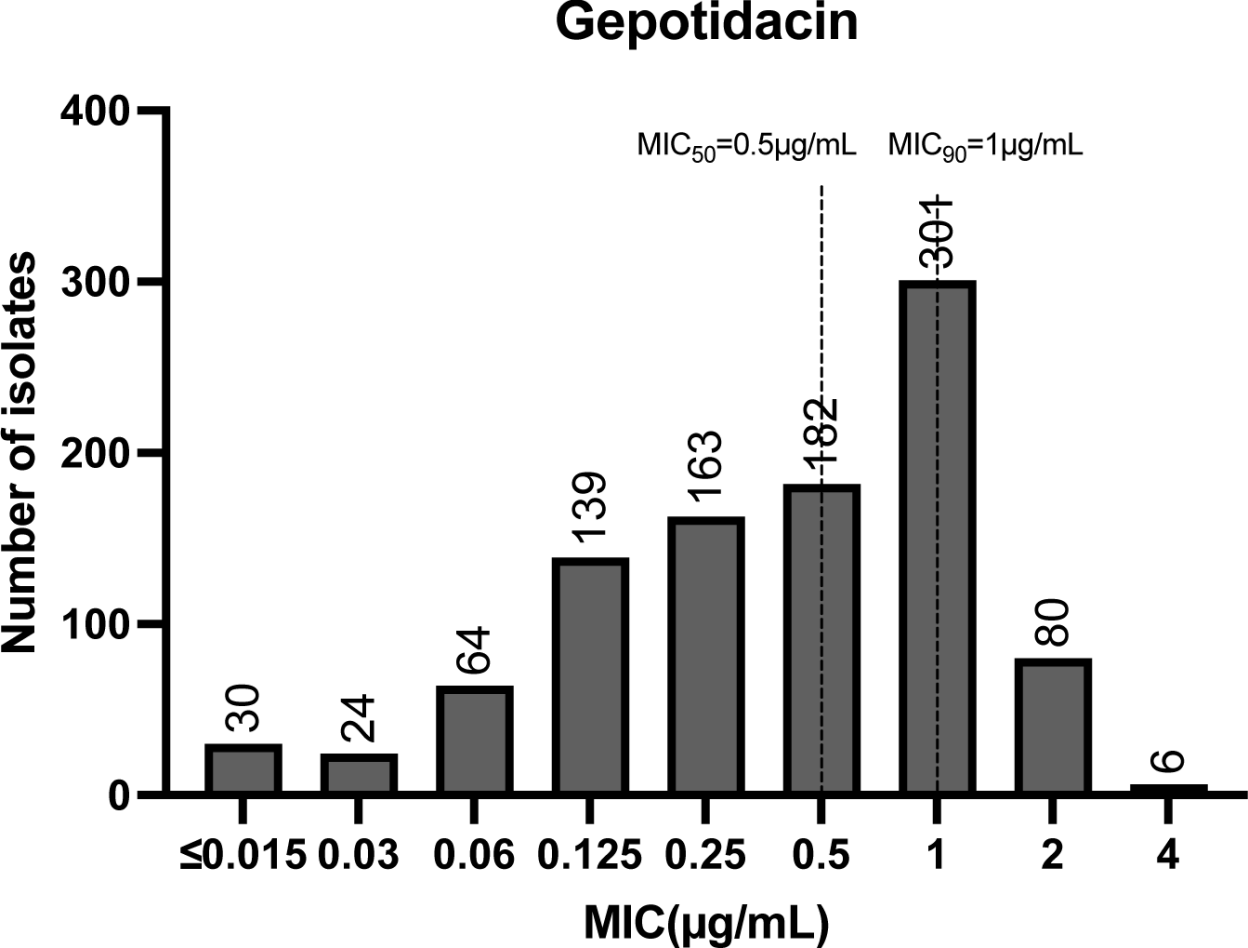
Gepotidacin MIC distribution of 989 *N. gonorrhoeae* isolates. MIC_50_ and MIC_90_ were marked by the dashed lines. The number of isolates is indicated on the top of the bars.

Gepotidacin exhibited high *in vitro* activity against the collected isolates, with MICs ranging from ≤0.015 to 4 μg/mL and MIC_50_ and MIC_90_ values of 0.5 and 1 μg/mL, respectively (Fig. 1). This activity was maintained against isolates resistant or non-susceptible to ceftriaxone, azithromycin, and ciprofloxacin (Table 2). No significant differences in gepotidacin susceptibility were observed based on patient sex, age, anatomical site of isolation, or geographic location (Table 1), indicating that reduced susceptibility is not driven by demographic or clinical factors.

**TABLE 2:** Minimum inhibitory concentration distribution of gepotidacin in drug-resistant *N. gonorrhoeae* isolates^*a*^

Isolates	MIC (μg/mL)									MIC_50_ (μg/mL)	MIC_90_ (μg/mL)	MIC range (μg/mL)	*P* value
	≤0.015	0.03	0.06	0.125	0.25	0.5	1	2	4				
CRO-NS isolates (*n* = 99)	0	0	8	29	22	18	17	5	0	0.25	1	0.06–2	0.001
AZI-NS isolates (*n* = 94)	1	0	1	18	16	18	26	13	1	0.5	2	≤0.015–4	0.087
CIP-R isolates (*n* = 988)	30	24	64	139	162	182	301	80	6	0.5	1	≤0.015–4	0.577
MDR isolates (*n* = 16)	0	0	0	7	3	3	3	0	0	0.25	1	0.125–1	0.106

^*a*^ MIC: minimum inhibitory concentration; CRO-NS: ceftriaxone-non-susceptible; AZI-NS: azithromycin-non-susceptible; CIP-R: ciprofloxacin-resistant; MDR: multidrug-resistant, non-susceptible to ceftriaxone and azithromycin, and resistant to ciprofloxacin. Wilcoxon tests were used to calculate the *P* value.

### Individual GyrA or ParC substitutions associated with elevated gepotidacin MICs

**Fig 2:**
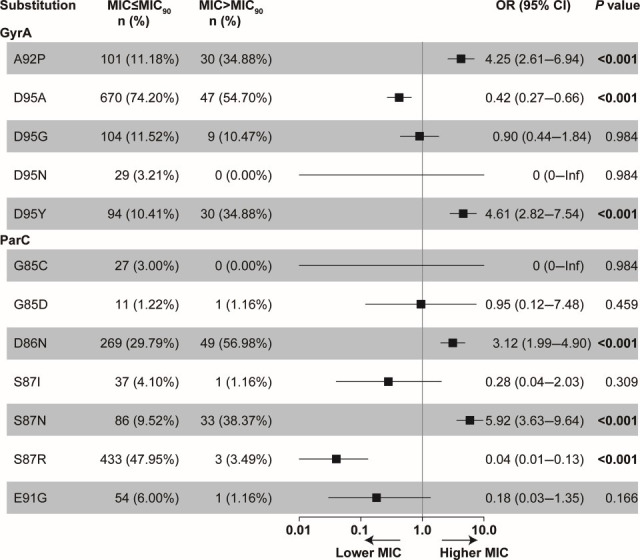
Association of individual GyrA and ParC substitutions with gepotidacin MICs. Univariate regression analysis was used for the test, and the substitutions with frequencies less than 1% or greater than 99% were not analyzed. OR, odds ratio; CI, confidence interval; NA, not available. The substitutions with isolates below 10 were listed in the Table S1.

To explore the genetic basis of reduced gepotidacin susceptibility, we analyzed QRDR mutations in GyrA and ParC across the 989 isolates. Nine substitutions were identified in GyrA and 16 in ParC (Fig. 2; Table S1). The most prevalent were S91F (99.9%, 988/989) and D95A (72.5%, 717/989) in GyrA, and S87R (44.1%, 436/989) and D86N (32.2%, 318/989) in ParC. Univariate logistic regression identified six substitutions significantly associated with gepotidacin MICs above or below the MIC_90_ (Fig. 2). GyrA A92P (OR, 4.25; 95% CI, 2.61–6.94) and D95Y (OR, 4.61; 95% CI, 2.82–7.54), as well as ParC D86N (OR, 3.12; 95% CI, 1.99–4.90) and S87N (OR, 5.92; 95% CI, 3.63–9.64), are linked to elevated MICs (*P* < 0.001). Conversely, GyrA D95A (OR, 0.42; 95% CI, 0.27–0.66) and S87R (OR, 0.04; 95% CI, 0.01–0.13) are associated with lower MICs (*P* < 0.001).

### Combined GyrA-ParC substitutions correlated with reduced gepotidacin susceptibility

Given gepotidacin’s dual-target action on GyrA and ParC (19), we further examined associations between combinations of GyrA and ParC substitutions and gepotidacin susceptibility. In total, we identified 43 GyrA-ParC substitution combinations (Table 3; Table S2). The most frequent combinations included GyrA D95A+ParC S87R (31.9%, 315/989), GyrA D95A+ParC D86N (30.0%, 297/989) and GyrA A92P/D95Y+ParC S87N (8.4%, 83/989). Nearly all isolates (99.9%, 988/989) carried GyrA S91F. Four combinations were significantly associated with elevated gepotidacin MICs: GyrA D95A+ParC D86N, GyrA A92P/D95Y+ParC S87N, GyrA D95A+ParC S87N, and GyrA D95G+ParC D86N (Table 3). These findings highlight the cooperative roles of GyrA codons 92/95 and ParC codons 86/87 in modulating gepotidacin activity and underscore the importance of combined mutations in driving reduced susceptibility.

**TABLE 3:** Gepotidacin MIC distribution of *N. gonorrhoeae* isolates with different GyrA-ParC substitution combinations^*a*^

GyrA	ParC	No.	No. of isolates with MIC (μg/mL)									MIC_50_ (μg/mL)	MIC_90_ (μg/mL)	MIC range (μg/mL)	*P* value	Effect
			≤0.015	0.03	0.06	0.125	0.25	0.5	1	2	4					
D95A	D86N	297	7	3	6	11	13	44	162	50	1	1	2	≤0.015–4	<0.001	Higher gepotidacin MIC
A92P, D95Y	S87N	83	0	2	2	4	2	7	36	28	2	1	2	0.03–4	<0.001	
D95A	S87N	29	0	0	1	1	3	9	11	3	1	1	2	0.06–4	0.014	
D95G	D86N	16	0	0	1	1	2	4	1	5	2	0.5	4	0.06–4	0.023	
D95A	S87R	315	16	9	38	85	85	49	32	1	0	0.25	1	≤0.015–2	<0.001	Lower gepotidacin MIC
D95G	S87R	53	1	3	7	13	12	11	5	1	0	0.25	1	≤0.015–2	<0.001	
D95N	S87I	12	1	0	0	3	4	4	0	0	0	0.25	0.5	≤0.015–0.5	0.042	
D95G	E91G	34	0	0	2	2	8	12	9	1	0	0.5	1	0.06–2	0.857	No difference
A92P, D95Y	S87R	19	1	0	0	5	4	5	4	0	0	0.25	1	≤0.015–1	0.207	
A92P, D95Y	S87I	15	0	0	0	1	6	4	4	0	0	0.5	1	0.125–1	0.893	
D95A	G85C	13	0	0	0	1	2	8	2	0	0	0.5	1	0.125–1	0.977	

^*a*^ GyrA S91F substitutions were presented in all these isolates. Wilcoxon tests were used to calculate the *P* values. The substitutions with isolates below 10 were listed in the Table S2.

### Clonal enrichment of high-risk GyrA-ParC substitution combinations in ST7363 and ST8123 lineages

**Fig 3:**
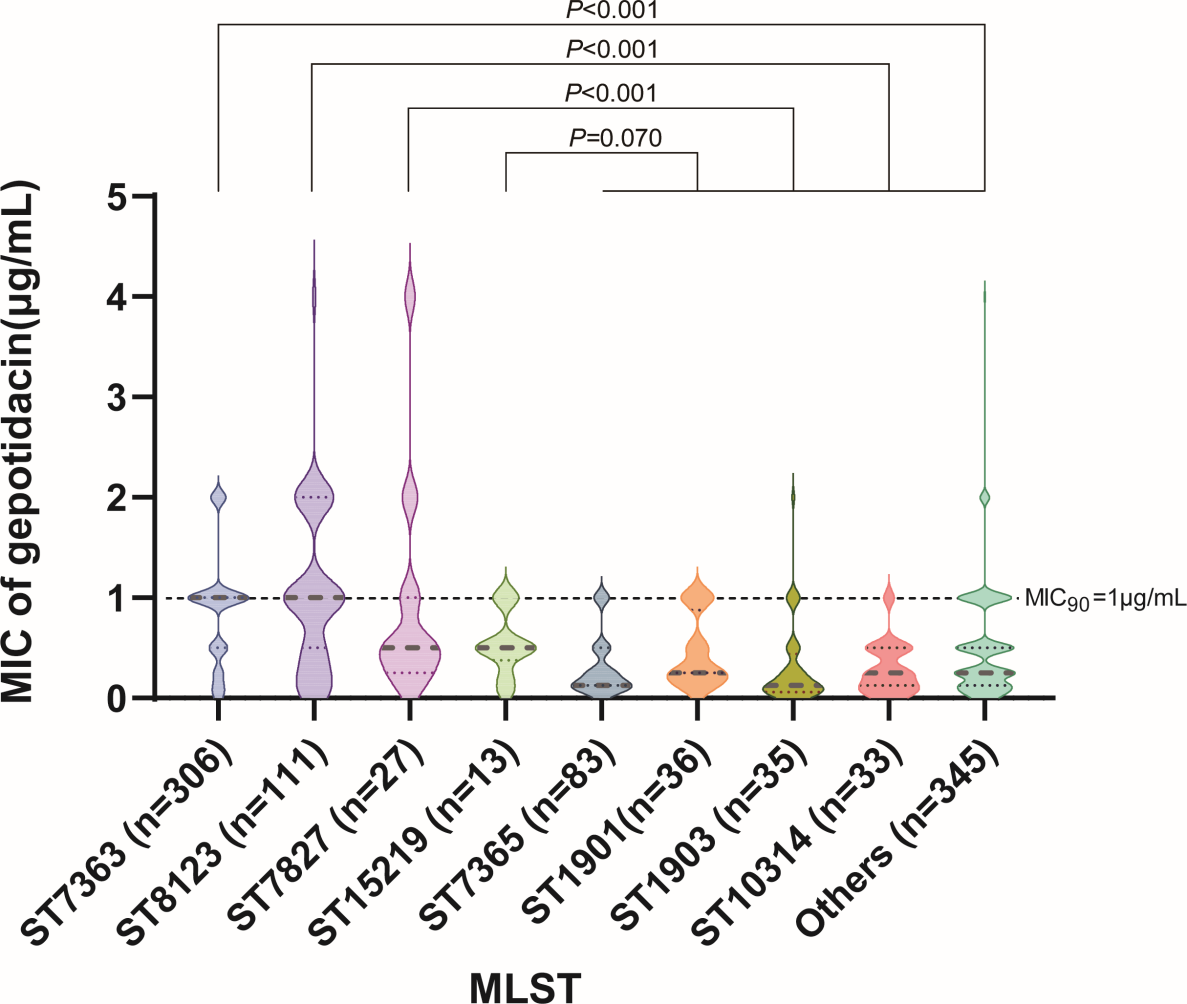
Distribution of gepotidacin MICs among major *N. gonorrhoeae* sequence types. Violin plots illustrate MIC variations in ST7363, ST8123, and all other STs. *P* values were calculated using Wilcoxon rank-sum tests.

Analysis of MLST types revealed striking clonal enrichment of high-risk QRDR combinations. Among isolates with GyrA D95A+ParC D86N, 85.9% (255/297) belonged to ST7363. Similarly, 90.5% (76/83) of isolates with GyrA A92P/D95Y+ParC S87N belonged to ST8123 (Table 4). MIC comparisons confirmed that these lineages exhibited significantly higher gepotidacin MICs than other sequence types (Fig. 3). These results suggest that ST7363 and ST8123 represent clonal backgrounds enriched for QRDR-associated point mutations and may facilitate the dissemination of reduced susceptibility through clonal expansion.

**TABLE 4:** Clonal distribution of GyrA-ParC substitution combinations associated with reduced gepotidacin susceptibility in *N. gonorrhoeae*›

GyrA^*a*^	ParC	No.	Major clone
D95A	D86N	297	ST7363 (255, 85.9%)
A92P, D95Y	S87N	83	ST8123 (76, 90.5%)
D95A	S87N	29	ST15219 (12, 41.4%)
D95G	D86N	16	ST7827 (8, 50.0%)

^*a*^ GyrA S91F substitutions were presented in all these isolates.

### *In vitro* induction experiments reveal a stepwise resistance trajectory from permissive QRDR mutations to high-level gepotidacin resistance

**Fig 4:**
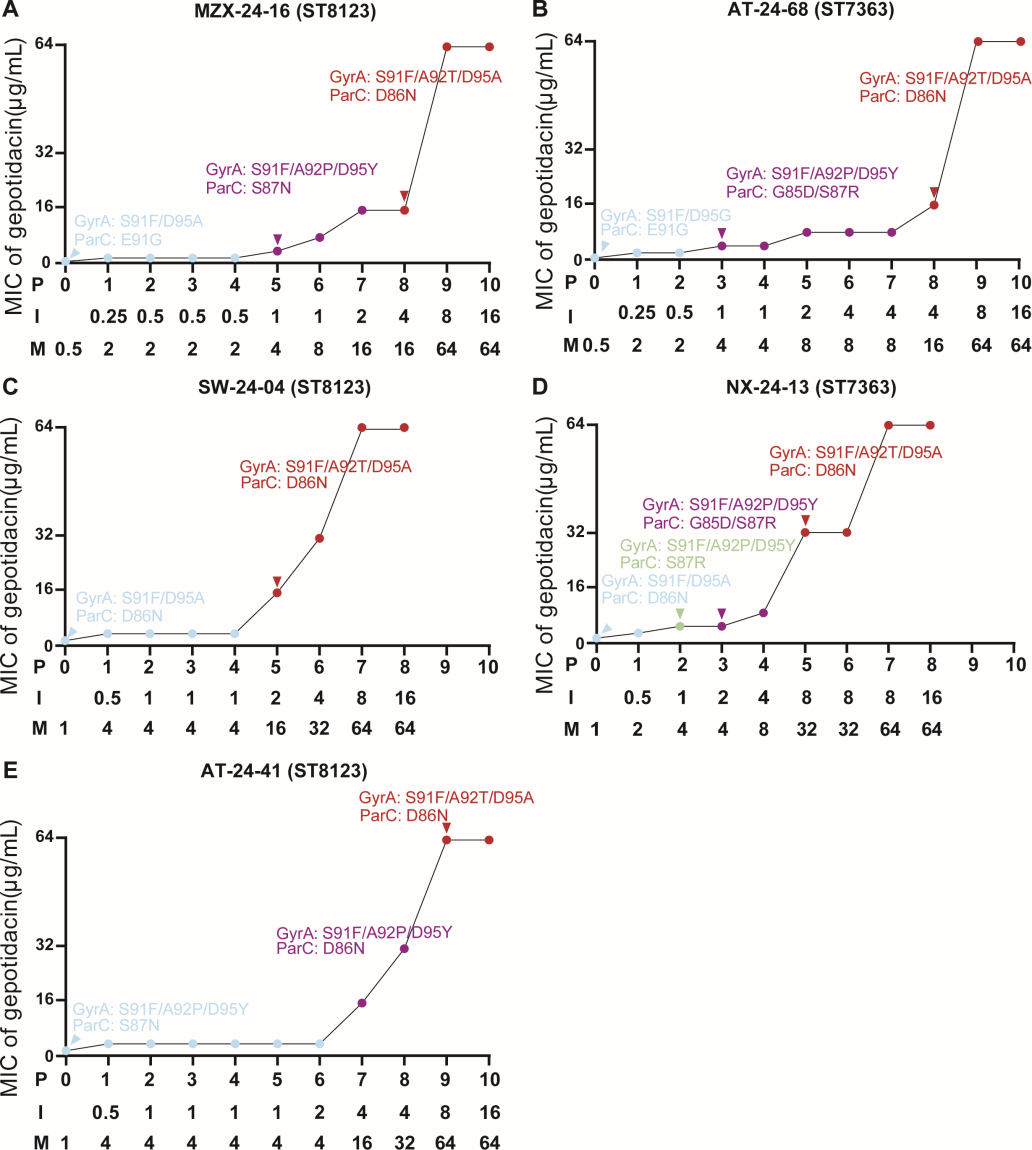
*In vitro* induction of gepotidacin resistance in *N. gonorrhoeae* ST8123 and ST7363 isolates. (**A**) MZX-24-16 (ST8123 isolate with GyrA S91F/D95A and ParC E91G). (**B**) AT-24-68 (ST7363 isolate with GyrA S91F/D95G and ParC E91G). (**C**) SW-24-04 (ST8123 isolate with GyrA S91F/D95A and ParC D86N). (**D**) NX-24-13 (ST7363 isolate with GyrA S91F/D95A and ParC D86N). (**E**) AT-24-41 (ST8123 isolate with GyrA S91F/A92P/D95Y and ParC S87N). The dots with the same color harbored the same QRDR mutation combinations in each panel. P: passages; I: inducing concentration (μg/mL); M: gepotidacin MIC (μg/mL).

To investigate the evolutionary pathway leading to high-level gepotidacin resistance, we performed *in vitro* induction assays using five gepotidacin-susceptible (MICs ≤ 1 µg/mL) clinical isolates of ST7363 and ST8123, each harboring distinct QRDR backgrounds. Upon serial passaging in sub-inhibitory concentrations of gepotidacin, all strains developed high-level resistance (MIC = 64 µg/mL) within 7–10 days (Fig. 4). Early in the induction process, isolates acquired GyrA A92P and D95Y along with ParC D86N or S87N, resulting in low-level resistance (MIC ≤ 8 µg/mL). With continued exposure, GyrA A92P consistently evolved into A92T, GyrA D95Y shifted to D95A, and ParC mutations converged on D86N, ultimately conferring high-level resistance (MIC ≥ 16 µg/mL).

Importantly, many of these intermediate mutational states, particularly GyrA A92P, D95Y and ParC D86N, S87N, were also enriched in clinical isolates belonging to ST7363 and ST8123, suggesting that these high-risk clones are genetically predisposed to evolve resistance through similar trajectories. Moreover, strains with preexisting GyrA S91F/D95A and ParC D86N backgrounds progressed to high-level resistance more rapidly than other strains (5 days vs 8–9 days), primarily through acquisition of GyrA A92T/D95A in the presence of ParC D86N, whereas other strains typically accumulated alternative QRDR substitutions (e.g., GyrA A92P or D95Y with ParC S87N, or GyrA D95G/D95A with ParC E91G) prior to reaching high-level resistance, underscoring the elevated evolutionary potential of this background under gepotidacin selection. These findings establish a defined stepwise resistance pathway in *N. gonorrhoeae* and reinforce the need for genomic surveillance strategies focused not only on individual mutations but on their sequential combinations, particularly in globally expanding lineages at heightened risk of resistance emergence.

## DISCUSSION

The global rise of gonococcal resistance has outpaced the development of new therapies. Gepotidacin, a promising dual-target antibiotic, offers renewed hope for treating multidrug-resistant *N. gonorrhoeae* (14, 20, 21), yet early indicators of resistance have already emerged (18). In this large-scale genomic and phenotypic study, we analyzed 989 clinical isolates from Shanghai and conducted targeted *in vitro* induction experiments. Our data reveal a clear association between substitutions within QRDRs of GyrA and ParC and reduced gepotidacin susceptibility, with these substitutions clonally enriched in globally expanding lineages such as ST7363 and ST8123. Moreover, our induction experiments recapitulated a stepwise evolutionary trajectory under gepotidacin pressure, consistent with pathways implicated in clinical trial failures. Together, these findings delineate the current resistance landscape and provide a molecular framework for anticipating future resistance evolution.

Our study revealed that gepotidacin retains high *in vitro* activity against clinical *N. gonorrhoeae* isolates, with MIC_50_ and MIC_90_ values estimated at 0.5 μg/mL and 1 μg/mL, consistent with previous reports (14, 22). We further identified positive associations between elevated gepotidacin MICs and specific QRDR substitutions, particularly in ParC D86N/S87N and GyrA A92P/D95A. In contrast, other substitutions, such as GyrA D95A and ParC S87R, showed inverse correlations with MICs, suggesting that the impact of QRDR variants on gepotidacin susceptibility is context-dependent rather than uniformly directional. Interestingly, we found a negative association between gepotidacin MICs and ceftriaxone susceptibility. Further analysis revealed that substitutions associated with reduced gepotidacin susceptibility (GyrA D95A plus ParC D86N, GyrA A92P/D95Y plus ParC S87N, GyrA D95A plus ParC S87N, and GyrA D95G plus ParC D86N) were overrepresented among ceftriaxone-susceptible isolates (46.4%, 413/890) compared with ceftriaxone-non-susceptible isolates (19.2%, 19/99). These findings are consistent with a recent study, which also reported an overrepresentation of ParC D86N among ceftriaxone-susceptible isolates (37.8%) compared with ceftriaxone-resistant isolates (13.8%) (22). The lower gepotidacin MICs observed in ceftriaxone-non-susceptible isolates may have clinical implications and warrant further investigation.

Beyond single substitutions, we explored the phenotypic effects of specific GyrA-ParC substitution combinations, including GyrA D95A+ParC D86N, GyrA A92P/D95Y+ParC S87N, GyrA D95A+ParC S87N, and GyrA D95G+ParC D86N, each of which was significantly associated with higher gepotidacin MICs. A prior structural study predicted that these residues are located near the gepotidacin-binding cleft of the DNA gyrase–topoisomerase IV complex, where polarity changes may weaken drug-target interactions, providing a structural-mechanistic explanation for the MIC elevation associated with QRDR mutations observed in this study (23). Our findings support close surveillance for such high-impact mutations, particularly in permissive backgrounds such as ParC D86N.

Building on these observations, we used *in vitro* induction to map the evolutionary pathway to gepotidacin resistance. At early passages, strains carrying preexisting GyrA S91F acquired A92P and D95Y together with ParC D86N, S87N, or G85D/S87R, yielding low-level resistance (MIC ≤ 8 µg/mL). With continued gepotidacin exposure, GyrA A92P converted to A92T, GyrA D95Y shifted to D95A, and ParC variants converged on D86N, ultimately producing high-level resistance (MIC ≥ 16 µg/mL). Notably, GyrA S91F persisted throughout induction, underscoring its central role in the resistance trajectory. This procedure mirrors clinical trial observations: ParC D86N was present at baseline in the Phase II failure case, whereas GyrA A92T emerged post-treatment (18), and our data provide supporting evidence for this stepwise evolution. Although A92T was not detected among our clinical isolates, it arose in every induced lineage *in vitro*, uniformly marking the transition from low- to high-level resistance and thereby underscoring its pivotal role in gepotidacin resistance. Notably, strains carrying the GyrA D95A+ParC D86N background more readily evolved high-level resistance, as shown by their earlier emergence of GyrA A92T relative to other genotypes. These findings highlight a specific, high-risk resistance axis that warrants focused monitoring in future surveillance.

Mutations associated with elevated gepotidacin MICs were not randomly distributed. Instead, they showed pronounced clonal clustering. Specifically, 85.9% (255/297) of isolates with ParC D86*N*+GyrA D95A belonged to ST7363, whereas 90.5% (76/83) of those with ParC S87*N*+GyrA D95Y/A92P belonged to ST8123. Consistent with this, ST7363 and ST8123 isolates exhibited elevated gepotidacin MICs. These patterns suggest that these clones may serve as genetic amplifiers of future gepotidacin resistance, highlighting the need for clone-aware surveillance that goes beyond tracking individual mutations to assess lineage-specific risk. Notably, MLST clones, including ST7363, ST8123, and ST1901, have been reported worldwide as ceftriaxone-resistant high-risk clones (8, 24–28). In this study, we found that these high-risk clones also exhibited higher gepotidacin MICs compared with other clones, implying that they tend to be multidrug-resistant, even to the newly developed agent gepotidacin, and that enhanced surveillance, particularly in these genetic backgrounds, is warranted. Our results further indicate that the use of gepotidacin for the clinical treatment of infections caused by these high-risk clones should be approached with caution and guided by antimicrobial susceptibility testing.

In addition to those mutations, our findings underscore drug exposure as a key driver of gepotidacin resistance. Prior hollow-fiber infection models show that subtherapeutic gepotidacin concentrations promote resistance (29). The MIC_90_ in our study (1 μg/mL) is consistent with reports from the WHO Enhanced Gonococcal Antimicrobial Surveillance Programme and with MIC values reported for isolates associated with treatment failure in Phase II clinical trials (18, 22). Pharmacokinetic data indicate that the current Phase III regimen, two oral doses of 3,000 mg administered 12 h apart, achieves plasma (*C*_max_ > 10 µg/mL) and urine (*C*_max_ > 200 µg/mL) concentrations well above this MIC_90_ (1 μg/mL) (15, 30). However, suboptimal drug exposure may still create a window for resistance selection. Notably, our induction assays demonstrated that sustained low-level exposure can drive stepwise resistance evolution, ultimately leading to high-level gepotidacin resistance (MIC = 64 µg/mL). This finding is particularly concerning given that gepotidacin has been approved in the United States for the treatment of uUTIs, where inappropriate use or suboptimal dosing, especially in the setting of unrecognized gonococcal co-infection, could potentially promote the emergence of resistance in *N. gonorrhoeae* (17). Together, these results underscore the critical need for exposure-optimized regimens when considering gepotidacin for the treatment of gonorrhea, uUTIs, or future indications. Ensuring adequate pharmacodynamic coverage across diverse patient populations and infection sites will be essential to safeguard the long-term clinical utility of this novel antimicrobial agent.

The ceftriaxone resistance rate in our study differed from those reported in other studies, which is largely attributable to the use of different interpretive guidelines and breakpoints. In this study, we applied the CLSI guidelines (35th edition), which define reduced susceptibility to ceftriaxone as an MIC > 0.25 µg/mL, resulting in a resistance rate of 10%. In contrast, studies from Vietnam conducted in 2023 and 2024 (28) and from China in 2022 (31) applied EUCAST breakpoints (MIC > 0.125 µg/mL) and reported resistance rates of 27% and 8.1%, respectively. If consistent breakpoints were applied, the ceftriaxone resistance rate in the present study would be 18%.

This study has several limitations. First, although QRDR mutations were considered to be associated with gepotidacin resistance, other potential mechanisms, such as efflux pump overexpression, may also contribute to reduced susceptibility. Second, although our data suggest that the combination of GyrA S91F/D95A/A92T and ParC D86N is linked to reduced gepotidacin susceptibility, definitive validation will require targeted allelic replacement in isogenic backgrounds. Third, the isolates included in this study were limited to the urogenital tract, as patients with pharyngeal or rectal gonorrhea were not routinely tested. Therefore, further studies focusing on pharyngeal and rectal infections are warranted, particularly given that microbiological failures at pharyngeal sites were reported in a previous study (15). Finally, the *in vitro*-induced mutations may not fully recapitulate the resistance mechanisms that emerge in clinical settings.

In conclusion, gepotidacin shows strong activity against clinical *N. gonorrhoeae* isolates, but reduced susceptibility or resistance can emerge through specific QRDR mutations, particularly within ST7363 and ST8123. Our *in vitro* induction experiments revealed a stepwise pathway to high-level resistance, highlighting the importance of optimized dosing and molecular surveillance to preserve gepotidacin’s clinical utility.

## MATERIALS AND METHODS

### *N. gonorrhoeae* strains isolation

A total of 989 *N. gonorrhoeae* isolates were collected from 33 hospitals participating in the Shanghai Gonococcal Resistance Surveillance Programme (SH-GRSP) between January 2022 and July 2024. The isolates were cultured at 37°C in a humidified environment with 5% CO₂ on agar plates. After subculturing, single colonies were selected and expanded, suspended in a homogeneous bacterial suspension, and stored at −80°C until further use. The study was approved by the Ethics Committee of Xinhua Hospital Affiliated to Shanghai Jiao Tong University School of Medicine (Reference number: No. XHEC-D-2025-164), and informed consent was waived.

### Antimicrobial susceptibility testing

Gepotidacin, ceftriaxone, azithromycin, and ciprofloxacin were purchased from MCE (MedChemExpress). The minimum inhibitory concentration (MIC) of *N. gonorrhoeae* to antimicrobial agents was determined using the agar dilution method, following the guidelines provided by the Clinical and Laboratory Standards Institute (CLSI). The *N. gonorrhoeae* ATCC49226 was used as the reference strain. The resistance breakpoints for ciprofloxacin (≥1 µg/mL), ceftriaxone (>0.25 µg/mL, non-susceptible), and azithromycin (>1 µg/mL, non-susceptible) were set based on CLSI (32). As there are no established breakpoints for gepotidacin, MIC values greater than the MIC_90_ were considered indicative of reduced susceptibility in this study.

### Whole-genome sequencing

Genomic DNA was extracted from all *N. gonorrhoeae* isolates using bacterial genomic DNA extraction kit (TIANGEN Biotech, Beijing, China) for whole-genome sequencing. Sequencing was performed on the Illumina HiSeq platform using 150 bp paired-end technology. The sequencing reads were assembled using SPAdes V3.8 (33) with default settings, excluding any contigs shorter than 500 nucleotides. Mutations in the QRDR of *gyrA* and *parC* were identified using pyngSTar with a database from CARD (34, 35). The clonal types were matched based on the pubMLST database (36).

### *In vitro* induction experiment

Gonorrhea strains were plated on agar supplemented with gepotidacin at 1/2 MIC and incubated overnight at 37°C in a 5% CO₂ atmosphere for 18–24 h. Upon observation of visible growth, a uniform bacterial suspension was adjusted to an OD_600_ of 0.08–0.085. A 100 μL aliquot of this suspension was then inoculated onto agar plates containing a gepotidacin concentration twice that of the preceding step. If growth was absent, the strain was propagated again on plates with the previous drug concentration. Bacteria from each passage were confirmed as *N. gonorrhoeae* through microscopic examination, gram staining, and oxidase testing. This process was repeated for each strain until *N. gonorrhoeae* capable of growing on plates containing 16 μg/mL gepotidacin was obtained. The MIC of each successive bacterial generation was determined using the agar dilution method. Genomic DNA was extracted from each generation, and PCR amplification was performed to detect resistance-associated mutations in the *gyrA* and *parC* genes using the following primers: *gyrA*-F: 5′-AACCCTGCCCGTCAGCCTTGA-3′, *gyrA*-R: 5′-GGACGAGCCGTTGACGAGCAG-3′, *parC*-F: 5′-GTTTCAGACGGCCAAAAGCC-3′, *parC*-R: 5′-GGCATAAAATCCACCGTCCCC-3′. Gene mutations were confirmed by Sanger sequencing.

### Statistical analysis

Substitutions with a frequency <1% or >99% were excluded. Univariate logistic regression was conducted to examine the association between GyrA and ParC substitutions and gepotidacin susceptibility. *P* values were adjusted using the Bonferroni correction. Chi-square (χ²) test was used to assess the differences in gender, age, isolation site, and geographic location. The MIC distributions of different groups were compared by using the Wilcoxon test. All *P* values were two-tailed, with a significance threshold of <0.05. All statistical analyses were performed in R software (version 4.4.1).

## Data Availability

The genome sequences of *N. gonorrhoeae* isolates have been deposited in the DDBJ/ENA/GenBank under project PRJNA956288. The GyrA and ParC substitutions used for the analyses are summarized in Table S3.

## SUPPLEMENTAL MATERIAL

Supplemental material aac.01706-25-s0001.docx Tables S1 to S3; Fig. S1.

ASM does not own the copyrights to Supplemental Material that may be linked to, or accessed through, an article. The authors have granted ASM a non-exclusive, world-wide license to publish the Supplemental Material files. Please contact the corresponding author directly for reuse.
