# Supplementary material for: Stepwise evolution and clonal enrichment of gepotidacin resistance in Neisseria gonorrhoeae
Source: Antimicrob Agents Chemother. 2026 Mar 11;70(4):e01706-25. doi: 10.1128/aac.01706-25 (PMC13041355; doi:10.1128/aac.01706-25)
Supplement: Supplemental material — Tables S1 to S3; Fig. S1. [file aac.01706-25-s0001.docx]

**Supplementary materials**

Table S1: GyrA or ParC substitutions with frequencies less than 1% or more than 99%

Table S2: Gepotidacin MIC distribution of *N. gonorrhoeae* isolates with different GyrA-ParC substitution combinations

Table S3: GyrA and ParC substitution combinations among all strains

Figure S1: Geographical distribution of source hospitals for 989 *N. gonorrhoeae* isolates in Shanghai

**Table S1. GyrA or ParC substitutions with frequencies less than 1% or more than 99%**

| **Substitution** | **All isolates (n=989)** | **Isolates with MIC≤MIC_90_ (n=903)** | **Isolates with MIC>MIC_90_ (n=86)** |
| --- | --- | --- | --- |
| GyrA | | | |
| V81I | 1 (0.1%) | 1 (0.1%) | 0 (0.0%) |
| S91F | 988 (99.9%) | 902 (99.9%) | 86 (100.0%) |
| S91I | 1 (0.1%) | 1 (0.1%) | 0 (0.0%) |
| N103D | 1 (0.1%) | 1 (0.1%) | 0 (0.0%) |
| ParC | | | |
| G85A | 2 (0.2%) | 2 (0.2%) | 0 (0.0%) |
| S87C | 7 (0.7%) | 7 (0.8%) | 0 (0.0%) |
| A89T | 7 (0.7%) | 7 (0.8%) | 0 (0.0%) |
| A89P | 1 (0.1%) | 1 (0.1%) | 0 (0.0%) |
| E91K | 1 (0.1%) | 1 (0.1%) | 0 (0.0%) |
| E91Q | 2 (0.2%) | 2 (0.2%) | 0 (0.0%) |
| G120E | 1 (0.1%) | 1 (0.1%) | 0 (0.0%) |
| G120R | 6 (0.6%) | 6 (0.7%) | 0 (0.0%) |
| A123P | 1 (0.1%) | 1 (0.1%) | 0 (0.0%) |
| Wild type | 7 (0.7%) | 7 (0.8%) | 0 (0.0%) |

**Table S2. Gepotidacin MIC distribution of *N. gonorrhoeae* isolates with different GyrA-ParC substitution combinations***

| **GyrA** | **ParC†** | **No.** | **MIC (μg/mL)** | | | | | | | | | **MIC_50_**  **(μg/mL)** | **MIC_90_**  **(μg/mL)** | **MIC range**  **(μg/mL)** | ***P* value** |
| --- | --- | --- | --- | --- | --- | --- | --- | --- | --- | --- | --- | --- | --- | --- | --- |
|  |  |  | **≤0.015** | **0.03** | **0.06** | **0.125** | **0.25** | **0.5** | **1** | **2** | **4** |  |  |  |  |
| D95N | S87R | 8 | 0 | 0 | 0 | 1 | 5 | 1 | 1 | 0 | 0 | 0.25 | 1 | 0.125-1 | 0.302 |
| D95A | E91G | 7 | 0 | 0 | 1 | 2 | 1 | 1 | 2 | 0 | 0 | 0.25 | 1 | 0.06-1 | 0.381 |
| D95A | S87I, E91G | 7 | 1 | 5 | 1 | 0 | 0 | 0 | 0 | 0 | 0 | 0.03 | 0.06 | ≤0.015-0.06 | <0.001 |
| D95A | S87C | 7 | 1 | 1 | 0 | 0 | 1 | 3 | 1 | 0 | 0 | 0.5 | 1 | ≤0.015-1 | 0.290 |
| D95A | G85C, S87R | 6 | 0 | 0 | 0 | 0 | 2 | 3 | 1 | 0 | 0 | 0.5 | 1 | 0.25-1 | 0.995 |
| D95A | G85D, S87R | 6 | 0 | 0 | 0 | 2 | 1 | 0 | 2 | 1 | 0 | 0.25 | 2 | 0.125-2 | 0.728 |
| D95A | Wild type | 6 | 0 | 0 | 0 | 0 | 2 | 1 | 3 | 0 | 0 | 0.5 | 1 | 0.25-1 | 0.484 |
| A92P, D95A | S87R | 6 | 0 | 0 | 2 | 0 | 2 | 2 | 0 | 0 | 0 | 0.25 | 0.5 | 0.06-0.5 | 0.108 |
| D95C | S87R | 5 | 0 | 0 | 0 | 1 | 2 | 2 | 0 | 0 | 0 | 0.25 | 0.5 | 0.125-0.5 | 0.348 |
| D95A | G85C, A89T | 4 | 0 | 0 | 0 | 0 | 0 | 2 | 2 | 0 | 0 | 0.5 | 1 | 0.5-1 | 0.326 |
| A92P, D95Y | G85D, S87R | 4 | 0 | 0 | 0 | 0 | 0 | 0 | 3 | 0 | 1 | 1 | 4 | 1-4 | 0.023 |
| D95N | D86N | 4 | 0 | 0 | 0 | 0 | 0 | 0 | 4 | 0 | 0 | 1 | 1 | 1-1 | 0.064 |
| D95N | E91G | 4 | 0 | 0 | 1 | 0 | 1 | 2 | 0 | 0 | 0 | 0.25 | 0.5 | 0.06-0.5 | 0.341 |
| D95A | S87R, G120R | 3 | 0 | 0 | 0 | 0 | 1 | 1 | 1 | 0 | 0 | 0.5 | 1 | 0.25-1 | 0.807 |
| D95G | S87R, G120R | 3 | 0 | 0 | 0 | 0 | 1 | 1 | 1 | 0 | 0 | 0.5 | 1 | 0.25-1 | 0.807 |
| D95A | S87N, E91G | 3 | 0 | 0 | 1 | 1 | 0 | 1 | 0 | 0 | 0 | 0.125 | 0.5 | 0.06-0.5 | 0.148 |
| D95G | S87R, G85D | 2 | 0 | 0 | 0 | 0 | 0 | 0 | 1 | 1 | 0 | 1 | 2 | 1-2 | 0.192 |
| D95A | S87I | 2 | 0 | 0 | 1 | 0 | 1 | 0 | 0 | 0 | 0 | 0.06 | 0.25 | 0.06-0.25 | 0.153 |
| D95A | S87N, E91Q | 2 | 0 | 0 | 0 | 0 | 1 | 1 | 0 | 0 | 0 | 0.25 | 0.5 | 0.25-0.5 | 0.725 |
| A92P, D95Y | G85C, A89T | 2 | 0 | 0 | 0 | 1 | 0 | 0 | 1 | 0 | 0 | 0.125 | 1 | 0.125-1 | 0.902 |
| A92P, D95Y | G85A, S87R | 1 | 0 | 0 | 0 | 1 | 0 | 0 | 0 | 0 | 0 | NA | NA | NA | NA |
| D95A | D86N, S87I | 1 | 0 | 0 | 0 | 0 | 0 | 0 | 0 | 1 | 0 | NA | NA | NA | NA |
| D95A | S87N, G120E | 1 | 0 | 0 | 0 | 0 | 0 | 0 | 1 | 0 | 0 | NA | NA | NA | NA |
| V81I, D95G | S87R | 1 | 0 | 1 | 0 | 0 | 0 | 0 | 0 | 0 | 0 | NA | NA | NA | NA |
| S91I, N103D | Wild type | 1 | 0 | 0 | 0 | 1 | 0 | 0 | 0 | 0 | 0 | NA | NA | NA | NA |
| D95G | G85A, S87R | 1 | 0 | 0 | 0 | 0 | 0 | 1 | 0 | 0 | 0 | NA | NA | NA | NA |
| A92P, D95A | G85C, S87R | 1 | 0 | 0 | 0 | 0 | 1 | 0 | 0 | 0 | 0 | NA | NA | NA | NA |
| D95G | S87I | 1 | 1 | 0 | 0 | 0 | 0 | 0 | 0 | 0 | 0 | NA | NA | NA | NA |
| D95A | S87R, A89P | 1 | 1 | 0 | 0 | 0 | 0 | 0 | 0 | 0 | 0 | NA | NA | NA | NA |
| D95N | S87N, E91K | 1 | 0 | 0 | 0 | 0 | 0 | 1 | 0 | 0 | 0 | NA | NA | NA | NA |
| D95G | G85C, A89T | 1 | 0 | 0 | 0 | 1 | 0 | 0 | 0 | 0 | 0 | NA | NA | NA | NA |
| D95G | S87R, A123P | 1 | 0 | 0 | 0 | 1 | 0 | 0 | 0 | 0 | 0 | NA | NA | NA | NA |

*****GyrA S91F substitutions were presented in all the isolates except for one isolate with GyrA S91I, GyrA N103D, and wild type ParC. Wilcoxon tests were used to calculate the *P* values. NA: not available.

**†**Wild type means no substitution was detected in QRDR regions of ParC

**Table S3. GyrA and ParC substitution combinations among all strains**

| ID | GyrA | ParC |
| --- | --- | --- |
| AD-22-02 | S91F, D95A | S87I, E91G |
| AD-23-01 | S91F, D95A | D86N |
| AD-23-04 | S91F, D95G | E91G |
| AD-23-06 | S91F, D95A | D86N |
| AT-22-08 | S91F, A92P, D95Y | S87N |
| AT-22-19 | S91F, D95A | E91G |
| AT-22-27 | S91F, D95A | S87R |
| AT-22-28 | S91F, A92P, D95Y | S87N |
| AT-22-29 | S91F, D95A | D86N |
| AT-22-30 | S91F, D95A | D86N |
| AT-22-35 | S91F, D95A | S87R |
| AT-22-36 | S91F, D95A | D86N |
| AT-22-37 | S91F, A92P, D95Y | S87N |
| AT-22-39 | S91F, D95A | S87R |
| AT-22-40 | S91F, D95G | S87R |
| AT-22-42 | S91F, D95A | D86N |
| AT-22-43 | S91F, D95N | S87I |
| AT-23-01 | S91F, D95A | G85D, S87R |
| AT-23-03 | S91F, A92P, D95Y | S87N |
| AT-23-06 | S91F, D95A | S87R |
| AT-23-08 | S91F, A92P, D95Y | S87N |
| AT-23-09 | S91F, D95A | D86N |
| AT-23-10 | S91F, A92P, D95Y | S87N |
| AT-23-21 | S91F, D95A | S87R |
| AT-23-23 | S91F, A92P, D95Y | S87N |
| AT-23-24 | S91F, D95A | D86N |
| AT-23-25 | S91F, D95A | D86N |
| AT-23-26 | S91F, D95A | S87R |
| AT-23-27 | S91F, D95N | S87R |
| AT-23-29 | S91F, D95A | D86N |
| AT-23-32 | S91F, D95A | S87R |
| AT-23-33 | S91F, D95N | E91G |
| AT-23-37 | S91F, D95A | S87R |
| AT-24-04 | S91F, D95A | S87R |
| AT-24-06 | S91F, A92P, D95Y | A89T, G85C |
| AT-24-07 | S91F, A92P, D95Y | S87N |
| AT-24-10 | S91F, D95A | D86N |
| AT-24-11 | S91F, D95A | D86N |
| AT-24-15 | S91F, D95A | S87R |
| AT-24-17 | S91F, D95A | S87R |
| AT-24-19 | S91F, D95A | S87R |
| AT-24-21 | S91F, D95G | S87R |
| AT-24-23 | S91F, D95G | S87R |
| AT-24-25 | S91F, A92P, D95Y | S87N |
| AT-24-26 | S91F, D95A | D86N |
| AT-24-27 | S91F, D95A | S87R |
| AT-24-28 | S91F, D95A | D86N |
| AT-24-29 | S91F, D95A | D86N |
| AT-24-32 | S91F, D95A | D86N |
| AT-24-33 | S91F, D95A | D86N |
| AT-24-35 | S91F, D95A | G120R, S87R |
| AT-24-41 | S91F, A92P, D95Y | S87N |
| AT-24-50 | S91F, D95N | S87R |
| AT-24-52 | S91F, D95A | D86N |
| AT-24-54 | S91F, A92P, D95Y | S87N |
| AT-24-55 | S91F, D95A | S87R |
| AT-24-58 | S91F, D95A | S87R |
| AT-24-60 | S91F, D95A | D86N |
| AT-24-62 | S91F, A92P, D95Y | S87R |
| AT-24-67 | S91F, D95A | D86N |
| AT-24-68 | S91F, D95G | E91G |
| AT-24-74 | S91F, D95A | D86N |
| AT-24-75 | S91F, A92P, D95Y | S87N |
| AT-24-76 | S91F, D95A | D86N |
| AT-24-77 | S91F, D95G | E91G |
| AT-24-78 | S91F, D95G | S87R |
| AT-24-79 | S91F, D95A | S87R |
| AT-24-80 | S91F, D95A | S87R |
| BD-23-01 | S91F, D95G | S87I |
| BD-23-03 | S91F, D95A | S87R |
| BD-23-04 | S91F, D95A | D86N |
| BD-23-05 | S91F, D95A | D86N |
| BD-23-07 | S91F, D95A | G85C |
| BD-23-08 | S91F, D95A | E91G |
| BD-23-10 | S91F, D95A | S87R |
| BD-23-11 | S91F, D95A | S87R |
| BD-23-112 | S91F, D95A | S87R |
| BD-23-12 | S91F, D95A | S87R |
| BD-23-122 | S91F, D95A | S87R |
| BD-23-13 | S91F, D95A | S87N |
| BD-23-14 | S91F, D95A | G85C |
| BD-23-15 | S91F, D95A | D86N |
| BD-23-16 | S91F, D95A | D86N |
| BD-23-17 | S91F, D95A | S87R |
| BD-23-19 | S91F, D95A | D86N |
| BD-23-20 | S91F, D95A | S87R |
| BD-23-21 | S91F, D95A | S87N |
| BD-23-22 | S91F, D95A | S87R |
| BD-23-23 | S91F, D95A | D86N |
| BD-23-24 | S91F, D95A | S87R |
| BD-23-25 | S91F, D95G | E91G |
| BD-23-26 | S91F, D95A | S87R |
| BD-23-29 | S91F, D95G | S87R |
| BD-23-31 | S91F, D95A | D86N |
| BD-23-32 | S91F, D95A | S87R |
| BD-23-33 | S91F, D95A | S87R |
| BD-23-34 | S91F, D95A | S87R |
| BD-23-35 | S91F, D95N | S87R |
| BD-23-36 | S91F, D95A | A89P, S87R |
| BD-23-37 | S91F, D95A | D86N, S87I |
| BD-23-38 | S91F, A92P, D95Y | S87N |
| BD-23-39 | S91F, A92P, D95Y | S87N |
| BD-23-41 | S91F, D95A | S87I, E91G |
| BD-23-42 | S91F, D95A | D86N |
| BD-23-43 | S91F, D95A | G85C |
| BD-23-45 | S91F, D95G | A89T, G85C |
| BD-23-46 | S91F, D95A | D86N |
| BD-23-47 | S91F, D95A | D86N |
| BD-23-48 | S91F, D95A | S87R |
| BD-23-49 | S91F, A92P, D95Y | S87R |
| BD-23-51 | S91F, D95A | S87R |
| BD-23-53 | S91F, D95A | S87R |
| BD-23-54 | S91F, D95G | S87R |
| BD-23-55 | S91F, D95A | S87N |
| BD-23-58 | S91F, D95A | G85C, S87R |
| BD-23-59 | S91F, D95A | S87R |
| BD-23-70 | S91F, A92P, D95Y | S87N |
| BD-23-71 | S91F, D95A | D86N |
| BD-23-80 | S91F, D95A | D86N |
| BD-23-84 | S91F, D95A | D86N |
| BD-23-85 | S91F, D95A | D86N |
| BD-23-86 | S91F, D95A | D86N |
| BD-23-89 | S91F, D95A | D86N |
| BD-24-06 | S91F, D95A | D86N |
| BD-24-07 | S91F, A92P, D95Y | S87N |
| BD-24-09 | S91F, A92P, D95Y | S87R |
| BD-24-10 | S91F, D95A | S87R |
| BD-24-11 | S91F, A92P, D95A | S87R |
| BD-24-12 | S91F, D95G | E91G |
| BD-24-13 | S91F, D95A | S87R |
| BD-24-14 | S91F, A92P, D95Y | S87N |
| BD-24-15 | S91F, D95A | D86N |
| BD-24-18 | S91F, D95A | S87R |
| BD-24-19 | S91F, A92P, D95Y | S87N |
| BD-24-20 | S91F, D95A | S87N |
| BD-24-21 | S91F, D95A | S87R |
| BD-24-22 | S91F, D95A | D86N |
| BD-24-24 | S91F, D95A | S87R |
| BD-24-25 | S91F, A92P, D95Y | S87I |
| BD-24-26 | S91F, D95A | S87R |
| BD-24-27 | S91F, A92P, D95Y | S87R |
| BD-24-28 | S91F, D95A | S87R |
| BD-24-29 | S91F, A92P, D95Y | S87R |
| BD-24-30 | S91F, D95G | S87R |
| BD-24-31 | S91F, D95A | S87R |
| BD-24-32 | S91F, D95A | S87R |
| BD-24-33 | S91F, D95A | S87R |
| BD-24-34 | S91F, D95G | S87R |
| BD-24-35 | S91F, D95G | D86N |
| BD-24-36 | S91F, D95A | D86N |
| BD-24-37 | S91F, D95G | S87R |
| BD-24-38 | S91F, D95G | S87R |
| BD-24-39 | S91F, A92P, D95Y | S87N |
| BD-24-40 | S91F, A92P, D95Y | S87N |
| BD-24-41 | S91F, D95A | D86N |
| BD-24-42 | S91F, D95A | S87R |
| BD-24-43 | S91F, D95A | S87R |
| BD-24-44 | S91F, D95A | G85C |
| BD-24-45 | S91F, D95A | S87N |
| BD-24-46 | S91F, D95G | S87R |
| BD-24-47 | S91F, D95G | E91G |
| BD-24-48 | S91F, D95G | E91G |
| BD-24-49 | S91F, D95G | S87R |
| BD-24-50 | S91F, D95A | S87R |
| BD-24-51 | S91F, A92P, D95Y | S87I |
| BD-24-52 | S91F, D95A | S87R |
| BD-24-53 | S91F, D95A | S87R |
| BD-24-54 | S91F, A92P, D95Y | A89T, G85C |
| BD-24-55 | S91F, A92P, D95Y | S87N |
| BD-24-56 | S91F, D95A | S87N |
| BD-24-57 | S91F, D95A | D86N |
| BD-24-58 | S91F, D95A | D86N |
| BD-24-59 | S91F, D95A | E91G |
| BD-24-60 | S91F, D95A | WT |
| BD-24-61 | S91F, D95A | S87R |
| BD-24-62 | S91F, D95A | S87R |
| BD-24-63 | S91F, D95A | S87R |
| BD-24-64 | S91F, D95A | S87R |
| BD-24-65 | S91F, D95A | S87R |
| BD-24-66 | S91F, D95C | S87R |
| BD-24-67 | S91F, D95A | S87R |
| BZX-24-01 | S91F, D95A | D86N |
| BZX-24-02 | S91F, D95A | S87R |
| BZX-24-03 | S91F, D95A | S87R |
| BZX-24-05 | S91F, D95A | S87R |
| BZX-22-01 | S91F, D95A | S87R |
| BZX-22-02 | S91F, D95A | D86N |
| BZX-22-03 | S91F, D95A | S87R |
| BZX-22-04 | S91F, A92P, D95Y | S87R |
| BZX-22-05 | S91F, D95A | S87R |
| BZX-22-06 | S91F, D95A | D86N |
| BZX-23-01 | S91F, D95A | D86N |
| BZX-23-02 | S91F, D95A | S87R |
| BZX-23-03 | S91F, A92P, D95Y | S87R |
| BZX-23-04 | S91F, D95A | S87R |
| BZX-23-05 | S91F, D95A | D86N |
| BZX-23-06 | S91F, D95A | D86N |
| BZX-23-07 | S91F, D95A | S87R |
| BZX-23-08 | S91F, D95A | S87R |
| BZX-23-09 | S91F, D95A | S87R |
| BZX-23-10 | S91F, D95A | S87R |
| BZX-23-11 | S91F, D95A | D86N |
| BZX-23-12 | S91F, D95A | S87N |
| BZX-23-15 | S91F, A92P, D95Y | S87N |
| BZY-23-02 | S91F, D95A | D86N |
| BZY-23-03 | S91F, D95A | S87R |
| BZY-23-04 | S91F, D95A | S87R |
| CE-22-01 | S91F, D95A | D86N |
| CE-22-07 | S91F, D95A | D86N |
| CE-22-08 | S91F, D95A | D86N |
| CE-22-09 | S91F, D95A | D86N |
| CE-22-10 | S91F, D95A | D86N |
| CE-22-12 | S91F, D95A | D86N |
| CE-22-13 | S91F, D95A | D86N |
| CE-22-15 | S91F, D95A | D86N |
| CE-22-16 | S91F, D95A | D86N |
| CE-22-18 | S91F, D95A | S87R |
| CE-22-19 | S91F, D95A | D86N |
| CE-23-01 | S91F, A92P, D95Y | S87N |
| CE-23-02 | S91F, D95A | D86N |
| CE-23-09 | S91F, D95A | D86N |
| CE-24-09 | S91F, D95A | S87R |
| CE-24-12 | S91F, D95A | D86N |
| CE-24-17 | S91F, D95A | D86N |
| CX-22-01 | S91F, D95A | D86N |
| CX-22-02 | S91F, D95A | S87R |
| CX-22-03 | S91F, D95A | D86N |
| CX-22-04 | S91F, D95N | E91G |
| CX-22-05 | S91F, D95A | G85C |
| CX-22-06 | S91F, D95A | D86N |
| CX-22-08 | S91F, D95N | E91G |
| CX-22-10 | S91F, D95N | D86N |
| CX-22-12 | S91F, D95A | D86N |
| CX-22-14 | S91F, D95A | S87R |
| CX-22-19 | S91F, D95A | D86N |
| CX-22-21 | S91F, D95A | S87R |
| CX-22-26 | S91F, D95A | D86N |
| CX-22-27 | S91F, D95A | D86N |
| CX-22-29 | S91F, D95A | S87R |
| CX-22-30 | S91F, D95A | D86N |
| CX-22-32 | S91F, D95A | S87R |
| CX-22-33 | S91F, D95A | D86N |
| CX-22-34 | S91F, D95A | D86N |
| CX-22-35 | S91F, D95A | D86N |
| CX-22-37 | S91F, D95A | D86N |
| CX-22-39 | S91F, D95A | D86N |
| CX-22-41 | S91F, D95G | D86N |
| CX-22-42 | S91F, D95G | D86N |
| CX-23-01 | S91F, D95A | D86N |
| CX-23-02 | S91F, A92P, D95Y | S87N |
| CX-23-03 | S91F, D95A | D86N |
| CX-23-04 | S91F, A92P, D95Y | S87N |
| CX-23-07 | S91F, D95A | D86N |
| CX-23-08 | S91F, D95A | S87R |
| CX-23-10 | S91F, D95A | S87R |
| CX-23-12 | S91F, D95A | S87R |
| CX-23-13 | S91F, D95A | S87R |
| CX-23-14 | S91F, D95A | D86N |
| CX-23-15 | S91F, D95A | D86N |
| CX-23-16 | S91F, D95A | D86N |
| JDZX-22-01 | S91F, D95A | S87R |
| JDZX-22-02 | S91F, D95A | D86N |
| JDZX-22-03 | S91F, D95A | S87R |
| JDZX-22-04 | S91F, D95C | S87R |
| JDZX-22-05 | S91F, D95A | S87R |
| JDZX-22-06 | S91F, D95A | D86N |
| JDZX-22-07 | S91F, D95A | S87R |
| JDZX-22-08 | S91F, D95A | S87R |
| JDZX-22-09 | S91F, D95A | D86N |
| JDZX-22-10 | S91F, D95A | A89T, G85C |
| JDZX-22-11 | S91F, A92P, D95Y | S87R |
| JDZX-22-12 | S91F, A92P, D95Y | S87N |
| JDZX-23-01 | S91F, D95A | S87R |
| JDZX-23-02 | S91F, D95C | S87R |
| JDZX-23-03 | S91F, A92P, D95Y | S87R |
| JDZX-23-06 | S91F, D95A | S87R |
| JDZX-23-07 | S91F, D95A | S87R |
| JDZX-23-08 | S91F, D95A | S87R |
| JDZX-23-09 | S91F, D95A | E91G |
| JDZX-23-10 | S91F, D95A | D86N |
| JDZX-23-11 | S91F, A92P, D95Y | S87N |
| JDZX-23-12 | S91F, D95A | S87R |
| JDZX-23-13 | S91F, D95G | S87R |
| JDZX-23-14 | S91F, D95A | D86N |
| JDZX-23-15 | S91F, D95A | D86N |
| JDZX-23-17 | S91F, D95A | D86N |
| JDZX-23-19 | S91F, D95N | S87I |
| JDZX-23-20 | S91F, D95A | S87R |
| JDZX-23-21 | S91F, D95A | D86N |
| JDZX-23-25 | S91F, A92P, D95Y | G85D, S87R |
| JDZX-24-02 | S91F, D95A | S87R |
| JDZX-24-03 | S91F, A92P, D95Y | S87N |
| JDZX-24-06 | S91F, D95G | S87R |
| JDZX-24-08 | S91F, D95A | WT |
| JDZX-24-09 | S91F, D95A | S87R |
| JDZX-24-12 | S91F, D95A | D86N |
| JDZX-24-13 | S91F, D95G | E91G |
| JDZX-24-14 | S91F, A92P, D95Y | S87N |
| JDZX-24-15 | S91F, D95A | S87R |
| JDZX-24-16 | S91F, A92P, D95Y | S87I |
| JDZX-24-17 | S91F, D95A | D86N |
| JDZX-24-20 | S91F, D95A | S87R |
| JDZX-24-24 | S91F, D95A | S87R |
| JDZX-24-25 | S91F, D95A | S87R |
| JDZX-24-28 | S91F, D95G | S87R |
| JDZX-24-29 | S91F, A92P, D95Y | S87N |
| JDZX-24-32 | S91F, D95A | D86N |
| JDZX-24-38 | S91F, A92P, D95Y | S87N |
| JDZX-24-41 | S91F, A92P, D95A | S87R |
| JDZX-24-56 | S91F, D95A | D86N |
| JDZX-24-58 | S91F, D95A | D86N |
| JDZX-24-62 | S91F, D95G | D86N |
| JDZX-24-63 | S91F, D95A | S87R |
| JDZX-24-66 | S91F, A92P, D95Y | S87I |
| JDZX-24-67 | S91F, D95A | D86N |
| JDZX-24-68 | S91F, D95A | S87R |
| JDZX-24-69 | S91F, D95G | D86N |
| JDZX-24-70 | S91F, D95C | S87R |
| JDZX-24-71 | S91F, D95A | D86N |
| JDZX-24-73 | S91F, D95A | S87R |
| JDZX-24-74 | S91F, D95A | S87R |
| JDZX-24-75 | S91F, D95A | D86N |
| JDZX-24-76 | S91F, A92P, D95Y | S87I |
| JDZX-24-77 | S91F, A92P, D95Y | S87N |
| JDZX-24-78 | S91F, D95A | S87C |
| JDZX-24-79 | S91F, D95G | S87R |
| JDZX-24-80 | S91F, D95G | E91G |
| JDZX-24-81 | S91F, D95G | S87R |
| JDZX-24-83 | S91F, D95A | S87R |
| JDZX-24-84 | S91F, D95A | S87R |
| JS-22-03 | S91F, D95A | D86N |
| JS-22-04 | S91F, D95A | S87R |
| JS-22-05 | S91F, D95A | D86N |
| JS-22-06 | S91F, D95A | D86N |
| JS-22-07 | S91F, D95A | D86N |
| JS-22-08 | S91F, D95A | D86N |
| JS-22-09 | S91F, D95A | D86N |
| JS-22-11 | S91F, D95A | S87R |
| JS-22-12 | S91F, D95A | A89T, G85C |
| JS-22-13 | S91F, D95A | S87R |
| JS-22-14 | S91F, D95N | S87I |
| JS-22-15 | S91F, D95A | S87R |
| JS-22-16 | S91F, D95A | D86N |
| JS-22-17 | S91F, D95A | D86N |
| JS-23-01 | S91F, D95A | S87I |
| JS-23-02 | S91F, D95G | D86N |
| JS-23-03 | S91F, D95A | S87N |
| JS-23-04 | S91F, D95A | S87R |
| JS-23-07 | S91F, D95A | D86N |
| JS-23-08 | S91F, D95A | D86N |
| JS-23-09 | S91F, D95A | D86N |
| JS-23-10 | S91F, D95A | D86N |
| JS-23-12 | S91F, D95A | S87R |
| JS-23-16 | S91F, D95A | D86N |
| JS-23-17 | S91F, D95A | D86N |
| JS-23-18 | S91F, D95A | S87R |
| JS-23-20 | S91F, D95A | D86N |
| JS-23-21 | S91F, D95A | D86N |
| JS-23-25 | S91F, D95A | D86N |
| JS-23-27 | S91F, D95G | S87R |
| JS-23-28 | S91F, D95A | D86N |
| JS-23-29 | S91F, A92P, D95Y | S87I |
| JS-23-30 | S91F, D95A | D86N |
| JS-24-03 | S91F, D95A | D86N |
| JS-24-05 | S91F, D95A | D86N |
| JS-24-08 | S91F, D95A | S87R |
| JSL-23-05 | S91F, D95A | S87R |
| JSL-24-11 | S91F, D95A | S87R |
| JSL-24-15 | S91F, D95A | S87R |
| JSL-24-18 | S91F, D95A | D86N |
| JTL-22-01 | S91F, D95A | D86N |
| JTL-22-02 | S91F, D95A | D86N |
| JTL-22-03 | S91F, D95N | S87I |
| JTL-22-04 | S91F, D95A | S87R |
| JTL-22-05 | S91F, D95A | S87R |
| JZX-22-01 | S91F, D95A | D86N |
| JZX-22-02 | S91F, A92P, D95Y | S87N |
| JZX-22-03 | S91F, D95G | S87R |
| JZX-22-04 | S91F, D95A | D86N |
| JZX-22-06 | S91F, D95N | S87I |
| JZX-22-07 | S91F, D95A | S87R |
| JZX-22-08 | S91F, D95A | S87R |
| JZX-22-09 | S91F, D95G | S87R |
| JZX-22-10 | S91F, D95G | S87R |
| JZX-22-11 | S91F, D95A | S87R |
| JZX-23-03 | S91F, D95G | D86N |
| JZX-23-04 | S91F, A92P, D95Y | S87N |
| JZX-23-05 | S91F, D95A | S87R |
| JZX-23-06 | S91F, A92P, D95Y | S87N |
| MZJ-22-01 | S91F, D95A | D86N |
| MZJ-22-03 | S91F, D95A | D86N |
| MZJ-22-04 | S91F, D95A | D86N |
| MZJ-23-02 | S91F, D95A | D86N |
| MZJ-23-03 | S91F, D95A | D86N |
| MZJ-24-01 | S91F, D95A | D86N |
| MZJ-24-05 | S91F, D95A | S87R |
| MZJ-24-06 | S91F, D95A | S87R |
| MZX-22-01 | S91F, D95A | S87R |
| MZX-22-02 | S91F, D95A | D86N |
| MZX-22-03 | S91F, D95A | D86N |
| MZX-22-04 | S91F, D95N | S87I |
| MZX-22-05 | S91F, D95A | S87R |
| MZX-22-06 | S91F, D95A | G85C |
| MZX-22-07 | S91F, D95A | S87R |
| MZX-22-09 | S91F, D95A | S87N |
| MZX-22-10 | S91F, D95A | S87R |
| MZX-22-11 | S91F, D95A | D86N |
| MZX-22-12 | S91F, D95A | S87R |
| MZX-22-13 | S91F, D95A | D86N |
| MZX-23-01 | S91F, D95A | D86N |
| MZX-23-02 | S91F, A92P, D95Y | S87N |
| MZX-23-03 | S91F, D95A | D86N |
| MZX-23-04 | S91F, D95G | S87R |
| MZX-23-05 | S91F, D95A | D86N |
| MZX-23-06 | S91F, D95A | S87R |
| MZX-23-07 | S91F, D95A | S87R |
| MZX-23-08 | S91F, A92P, D95Y | S87N |
| MZX-23-09 | S91F, D95A | D86N |
| MZX-23-11 | S91F, D95A | S87R |
| MZX-23-12 | S91F, D95A | S87R |
| MZX-23-13 | S91F, D95A | S87C |
| MZX-23-14 | S91F, D95A | S87R |
| MZX-23-15 | S91F, D95G | S87R |
| MZX-23-16 | S91F, D95A | D86N |
| MZX-23-17 | S91F, D95G | E91G |
| MZX-23-18 | S91F, D95A | D86N |
| MZX-23-20 | S91F, D95A | D86N |
| MZX-23-21 | S91F, D95A | S87R |
| MZX-23-22 | S91F, D95A | D86N |
| MZX-23-23 | S91F, D95G | S87R |
| MZX-23-24 | S91F, D95A | S87R |
| MZX-23-25 | S91F, D95A | D86N |
| MZX-23-26 | S91F, A92P, D95Y | G85D, S87R |
| MZX-23-29 | S91F, D95A | G85D, S87R |
| MZX-23-30 | S91F, D95G | E91G |
| MZX-23-31 | S91F, D95A | S87R |
| MZX-23-32 | S91F, D95A | S87R |
| MZX-23-34 | S91F, D95A | S87R |
| MZX-23-36 | S91F, D95A | S87N |
| MZX-23-37 | S91F, D95A | D86N |
| MZX-24-01 | S91F, D95A | D86N |
| MZX-24-02 | S91F, D95A | S87R |
| MZX-24-03 | S91F, D95N | S87I |
| MZX-24-04 | S91F, D95A | S87R |
| MZX-24-05 | S91F, D95A | S87N |
| MZX-24-07 | S91F, D95G | S87R |
| MZX-24-08 | S91F, D95A | D86N |
| MZX-24-09 | S91F, D95G | E91G |
| MZX-24-11 | S91F, D95A | D86N |
| MZX-24-12 | S91F, D95G | D86N |
| MZX-24-14 | S91F, D95A | D86N |
| MZX-24-15 | S91F, D95A | D86N |
| MZX-24-16 | S91F, D95A | E91G |
| MZX-24-18 | S91F, D95A | S87R |
| MZX-24-19 | S91F, D95A | G85C |
| MZX-24-20 | S91F, D95A | D86N |
| MZX-24-21 | S91F, A92P, D95Y | S87N |
| MZX-24-22 | S91F, D95A | S87R |
| MZX-24-23 | S91F, D95A | D86N |
| MZX-24-25 | S91F, D95A | S87R |
| MZX-24-26 | S91F, D95A | D86N |
| MZX-24-27 | S91F, D95A | S87N |
| MZX-24-28 | S91F, D95A | S87R |
| MZX-24-29 | S91F, D95A | S87R |
| MZX-24-30 | S91F, D95G | S87R |
| MZX-24-31 | S91F, D95A | S87R |
| MZX-24-32 | S91F, D95A | D86N |
| MZX-24-33 | S91F, D95A | S87R |
| MZX-24-34 | S91F, D95N | S87I |
| NX-22-04 | S91F, D95A | D86N |
| NX-22-05 | S91F, D95A | D86N |
| NX-22-06 | S91F, D95A | S87R |
| NX-22-11 | S91F, D95A | S87R |
| NX-22-12 | S91F, D95A | D86N |
| NX-22-13 | S91F, D95A | S87R |
| NX-22-14 | S91F, D95A | S87N, E91G |
| NX-22-15 | S91F, D95A | D86N |
| NX-22-17 | S91F, D95A | D86N |
| NX-22-18 | S91F, D95A | S87R |
| NX-22-24 | S91F, D95A | S87R |
| NX-22-26 | S91F, D95A | S87R |
| NX-22-27 | S91F, D95A | S87R |
| NX-22-28 | S91F, D95A | S87R |
| NX-22-29 | S91F, D95A | S87R |
| NX-22-30 | S91F, D95N | E91K, S87N |
| NX-22-31 | S91F, D95A | D86N |
| NX-22-33 | S91F, D95G | S87R |
| NX-22-34 | S91F, D95G | S87R |
| NX-22-35 | S91F, A92P, D95Y | S87N |
| NX-22-37 | S91F, D95N | S87I |
| NX-22-38 | S91F, D95A | D86N |
| NX-22-39 | S91F, D95A | D86N |
| NX-22-40 | S91F, A92P, D95Y | S87R |
| NX-22-41 | S91F, D95A | D86N |
| NX-22-43 | S91F, D95A | S87R |
| NX-22-44 | S91F, D95A | S87N |
| NX-22-45 | S91F, D95A | S87R |
| NX-22-46 | S91F, D95A | D86N |
| NX-22-47 | V81I, S91F, D95G | S87R |
| NX-23-01 | S91F, D95A | D86N |
| NX-23-02 | S91F, A92P, D95Y | S87N |
| NX-23-03 | S91F, D95A | S87R |
| NX-23-04 | S91F, D95G | E91G |
| NX-23-05 | S91F, D95A | S87R |
| NX-23-06 | S91F, D95G | S87R |
| NX-23-07 | S91F, D95A | S87R |
| NX-23-08 | S91F, D95A | D86N |
| NX-23-10 | S91F, D95G | S87R |
| NX-23-11 | S91F, D95A | S87R |
| NX-23-12 | S91F, D95A | S87R |
| NX-23-13 | S91F, D95A | G120R, S87R |
| NX-23-14 | S91F, D95A | S87R |
| NX-23-15 | S91F, D95A | D86N |
| NX-23-16 | S91F, D95A | S87R |
| NX-23-17 | S91F, D95G | S87R |
| NX-23-18 | S91F, D95A | S87R |
| NX-23-19 | S91F, D95A | D86N |
| NX-23-20 | S91F, D95A | D86N |
| NX-23-22 | S91F, D95A | D86N |
| NX-23-27 | S91F, D95A | D86N |
| NX-23-28 | S91F, D95A | D86N |
| NX-23-29 | S91F, D95A | S87R |
| NX-23-30 | S91F, D95A | D86N |
| NX-23-31 | S91F, D95A | S87R |
| NX-23-32 | S91F, D95A | S87R |
| NX-23-35 | S91F, D95A | G120E, S87N |
| NX-23-39 | S91F, D95G | S87R |
| NX-23-43 | S91F, D95A | S87R |
| NX-23-44 | S91F, D95A | S87I, E91G |
| NX-23-45 | S91F, D95A | D86N |
| NX-23-46 | S91F, A92P, D95Y | S87N |
| NX-23-47 | S91F, A92P, D95Y | S87N |
| NX-24-01 | S91F, D95A | D86N |
| NX-24-02 | S91F, D95G | S87R |
| NX-24-03 | S91F, D95A | G85C |
| NX-24-04 | S91F, D95G | D86N |
| NX-24-05 | S91F, D95A | S87R |
| NX-24-06 | S91F, D95A | S87N |
| NX-24-07 | S91F, D95A | D86N |
| NX-24-09 | S91F, D95G | E91G |
| NX-24-10 | S91F, D95A | D86N |
| NX-24-11 | S91F, D95G | E91G |
| NX-24-12 | S91F, D95G | S87R |
| NX-24-13 | S91F, D95A | D86N |
| NX-24-14 | S91F, D95A | S87R |
| NX-24-15 | S91F, D95A | S87N |
| NX-24-16 | S91F, D95A | S87R |
| NX-24-17 | S91F, D95G | G120R, S87R |
| NX-24-19 | S91F, D95A | D86N |
| NX-24-20 | S91F, D95A | S87R |
| NX-24-21 | S91F, D95N | E91G |
| NX-24-22 | S91F, A92P, D95Y | S87R |
| NX-24-23 | S91F, D95G | E91G |
| NX-24-25 | S91F, D95G | G85D, S87R |
| NX-24-26 | S91F, D95A | WT |
| NX-24-27 | S91F, D95G | E91G |
| NX-24-28 | S91F, D95G | S87R |
| NX-24-31 | S91F, D95A | D86N |
| NX-24-32 | S91F, D95A | S87C |
| NX-24-33 | S91I, N103D | WT |
| NX-24-34 | S91F, D95A | D86N |
| NX-24-37 | S91F, D95A | S87R |
| NX-24-40 | S91F, D95A | S87R |
| NX-24-42 | S91F, D95A | S87R |
| NX-24-45 | S91F, A92P, D95Y | S87N |
| NX-24-46 | S91F, D95A | G85C |
| NX-24-49 | S91F, A92P, D95Y | S87N |
| NX-24-50 | S91F, A92P, D95A | S87R |
| NX-24-51 | S91F, A92P, D95Y | S87N |
| NX-24-53 | S91F, D95A | S87N |
| NX-24-54 | S91F, D95A | D86N |
| NX-24-55 | S91F, D95A | S87R |
| NX-24-56 | S91F, D95A | S87R |
| NX-24-58 | S91F, D95A | S87R |
| NX-24-60 | S91F, D95A | D86N |
| NX-24-61 | S91F, D95A | G120R, S87R |
| NX-24-63 | S91F, D95A | S87R |
| NX-24-64 | S91F, D95A | D86N |
| NX-24-65 | S91F, A92P, D95Y | S87R |
| NX-24-69 | S91F, D95G | E91G |
| NX-24-70 | S91F, A92P, D95Y | S87R |
| NX-24-71 | S91F, A92P, D95Y | S87R |
| NX-24-72 | S91F, D95G | S87R |
| NX-24-73 | S91F, D95A | S87R |
| NX-24-77 | S91F, D95A | G85D, S87R |
| NX-24-78 | S91F, D95A | S87R |
| NX-24-79 | S91F, D95A | S87R |
| NX-24-80 | S91F, A92P, D95Y | G85D, S87R |
| NX-24-81 | S91F, D95A | S87R |
| NX-24-87 | S91F, D95G | G85D, S87R |
| NX-24-91 | S91F, A92P, D95Y | S87N |
| NX-24-92 | S91F, D95A | S87R |
| NX-24-94 | S91F, A92P, D95Y | S87R |
| PD-23-01 | S91F, D95A | S87R |
| PD-23-05 | S91F, A92P, D95Y | S87I |
| PD-23-06 | S91F, D95A | S87R |
| PD-23-07 | S91F, D95A | S87R |
| PD-23-11 | S91F, D95A | D86N |
| PD-23-12 | S91F, D95A | S87R |
| PD-24-21 | S91F, A92P, D95Y | G85A, S87R |
| PD-24-23 | S91F, D95A | S87R |
| PD-24-24 | S91F, D95A | D86N |
| PD-24-25 | S91F, D95A | S87R |
| PD-24-28 | S91F, A92P, D95Y | S87N |
| PD-24-30 | S91F, D95A | D86N |
| PFY-23-01 | S91F, D95A | S87R |
| PFY-23-02 | S91F, D95A | S87R |
| PFY-23-03 | S91F, D95A | E91Q, S87N |
| PR-22-02 | S91F, D95A | D86N |
| PR-22-03 | S91F, D95N | S87I |
| PR-22-05 | S91F, D95A | D86N |
| PR-22-07 | S91F, D95A | S87R |
| PR-22-08 | S91F, D95N | S87R |
| PR-22-09 | S91F, D95A | A89T, G85C |
| PR-22-12 | S91F, D95A | D86N |
| PR-22-13 | S91F, D95N | S87I |
| PR-22-14 | S91F, A92P, D95Y | S87N |
| PR-22-15 | S91F, D95A | S87R |
| PR-22-16 | S91F, D95A | S87R |
| PR-22-17 | S91F, D95A | S87R |
| PR-22-18 | S91F, D95A | S87I, E91G |
| PR-22-19 | S91F, D95A | D86N |
| PR-22-20 | S91F, D95A | S87R |
| PR-22-22 | S91F, D95A | WT |
| PR-22-23 | S91F, D95A | S87R |
| PR-22-25 | S91F, D95A | D86N |
| PR-22-26 | S91F, D95A | S87R |
| PR-22-27 | S91F, A92P, D95Y | S87N |
| PR-22-28 | S91F, D95A | D86N |
| PR-22-29 | S91F, D95G | S87R |
| PR-22-30 | S91F, D95A | S87R |
| PR-22-33 | S91F, D95A | D86N |
| PR-22-36 | S91F, D95G | D86N |
| PR-22-37 | S91F, D95A | G85C, S87R |
| PR-22-39 | S91F, D95G | G120R, S87R |
| PR-22-40 | S91F, D95A | D86N |
| PR-22-41 | S91F, D95A | S87R |
| PR-22-42 | S91F, D95A | D86N |
| PR-22-45 | S91F, D95A | D86N |
| PR-22-46 | S91F, D95A | S87R |
| PR-23-01 | S91F, D95A | D86N |
| PR-23-03 | S91F, D95A | D86N |
| PR-23-04 | S91F, D95A | D86N |
| PR-23-05 | S91F, D95A | S87R |
| PR-23-06 | S91F, A92P, D95Y | S87N |
| PR-23-08 | S91F, A92P, D95Y | S87N |
| PR-23-10 | S91F, D95A | D86N |
| PR-23-12 | S91F, D95A | D86N |
| PR-23-13 | S91F, A92P, D95Y | S87N |
| PR-23-15 | S91F, D95G | S87R |
| PR-23-17 | S91F, D95A | D86N |
| PR-23-22 | S91F, D95A | D86N |
| PR-23-23 | S91F, A92P, D95Y | S87R |
| PR-24-02 | S91F, D95A | D86N |
| PR-24-05 | S91F, D95A | D86N |
| PR-24-22 | S91F, D95A | S87N |
| PR-24-23 | S91F, A92P, D95Y | S87N |
| PR-24-34 | S91F, D95G | A123P, S87R |
| QZF-23-02 | S91F, D95G | E91G |
| QZF-24-01 | S91F, D95A | G85C |
| SD-22-01 | S91F, D95A | S87C |
| SD-22-02 | S91F, D95A | D86N |
| SD-22-03 | S91F, D95A | S87N |
| SD-22-04 | S91F, D95A | S87R |
| SD-22-05 | S91F, A92P, D95Y | S87N |
| SD-22-06 | S91F, D95A | S87R |
| SD-22-07 | S91F, D95A | D86N |
| SD-23-01 | S91F, D95A | S87C |
| SD-23-02 | S91F, D95A | D86N |
| SD-23-03 | S91F, D95A | S87R |
| SD-23-04 | S91F, D95A | D86N |
| SD-23-05 | S91F, A92P, D95Y | S87N |
| SD-23-07 | S91F, D95A | S87N |
| SD-24-03 | S91F, D95A | S87R |
| SD-24-06 | S91F, A92P, D95A | S87R |
| SFB-22-05 | S91F, D95G | G85A, S87R |
| SFB-22-06 | S91F, D95A | D86N |
| SFB-22-08 | S91F, D95A | D86N |
| SFB-22-09 | S91F, D95A | S87R |
| SFB-24-06 | S91F, A92P, D95Y | S87N |
| SFB-24-07 | S91F, D95A | S87R |
| SL-22-01 | S91F, D95A | S87R |
| SL-22-02 | S91F, A92P, D95Y | S87N |
| SL-22-03 | S91F, D95A | S87I, E91G |
| SL-22-04 | S91F, A92P, D95Y | S87N |
| SL-22-05 | S91F, D95A | D86N |
| SL-22-06 | S91F, D95A | S87N |
| SL-22-07 | S91F, D95C | S87R |
| SL-22-09 | S91F, D95G | S87R |
| SL-22-10 | S91F, D95A | S87R |
| SL-22-13 | S91F, D95A | S87R |
| SL-22-17 | S91F, D95G | S87R |
| SL-22-18 | S91F, D95A | S87R |
| SL-22-23 | S91F, D95A | S87R |
| SL-22-25 | S91F, D95A | S87R |
| SL-22-26 | S91F, A92P, D95Y | S87R |
| SL-22-27 | S91F, D95A | A89T, G85C |
| SL-22-28 | S91F, D95A | G85D, S87R |
| SL-22-29 | S91F, D95A | S87R |
| SL-22-30 | S91F, A92P, D95Y | S87N |
| SL-22-31 | S91F, D95A | S87R |
| SL-23-01 | S91F, A92P, D95Y | S87N |
| SL-23-02 | S91F, D95G | S87R |
| SL-23-05 | S91F, D95A | D86N |
| SL-23-06 | S91F, D95G | S87R |
| SL-23-08 | S91F, D95A | D86N |
| SL-23-09 | S91F, D95A | WT |
| SL-23-10 | S91F, D95A | S87R |
| SL-23-11 | S91F, D95N | S87I |
| SL-23-12 | S91F, A92P, D95Y | S87N |
| SL-23-13 | S91F, D95A | D86N |
| SL-23-14 | S91F, D95A | S87R |
| SL-23-15 | S91F, D95G | E91G |
| SL-23-18 | S91F, D95A | D86N |
| SL-23-19 | S91F, D95A | D86N |
| SL-23-20 | S91F, D95A | D86N |
| SL-23-22 | S91F, A92P, D95Y | G85D, S87R |
| SL-23-23 | S91F, D95A | D86N |
| SL-23-24 | S91F, D95A | WT |
| SL-23-25 | S91F, D95G | E91G |
| SL-23-28 | S91F, A92P, D95Y | S87N |
| SL-23-29 | S91F, D95A | D86N |
| SL-23-30 | S91F, D95A | D86N |
| SL-23-31 | S91F, A92P, D95Y | S87N |
| SL-23-32 | S91F, D95A | S87R |
| SL-23-33 | S91F, D95A | S87R |
| SL-23-34 | S91F, D95G | E91G |
| SL-23-35 | S91F, D95G | S87R |
| SL-23-36 | S91F, D95A | D86N |
| SL-23-37 | S91F, D95A | S87R |
| SL-23-39 | S91F, D95A | D86N |
| SL-23-40 | S91F, D95A | S87R |
| SL-23-42 | S91F, D95N | D86N |
| SL-23-45 | S91F, D95A | E91G |
| SL-23-46 | S91F, D95A | S87R |
| SL-23-47 | S91F, D95A | G85C, S87R |
| SL-23-50 | S91F, D95A | S87N, E91G |
| SL-23-53 | S91F, D95A | S87R |
| SL-23-55 | S91F, D95G | E91G |
| SL-23-57 | S91F, D95A | S87N |
| SL-23-58 | S91F, D95A | S87R |
| SL-23-59 | S91F, D95A | D86N |
| SL-23-61 | S91F, D95G | S87R |
| SL-23-62 | S91F, A92P, D95Y | S87N |
| SL-24-02 | S91F, D95G | E91G |
| SL-24-05 | S91F, D95A | S87R |
| SL-24-08 | S91F, D95A | S87R |
| SL-24-09 | S91F, A92P, D95Y | S87N |
| SL-24-12 | S91F, D95A | S87R |
| SL-24-19 | S91F, D95A | S87N |
| SL-24-28 | S91F, A92P, D95Y | S87N |
| SL-24-32 | S91F, D95A | S87R |
| SL-24-33 | S91F, D95A | D86N |
| SL-24-34 | S91F, D95A | S87R |
| SL-24-35 | S91F, D95A | D86N |
| SL-24-36 | S91F, D95A | S87R |
| SL-24-42 | S91F, D95A | D86N |
| SQ-23-06 | S91F, D95A | S87R |
| SQ-24-04 | S91F, D95A | S87R |
| SQ-24-07 | S91F, D95A | S87N |
| SQ-24-08 | S91F, A92P, D95A | S87R |
| SQ-24-09 | S91F, D95A | D86N |
| SQ-24-10 | S91F, D95A | D86N |
| SQ-24-11 | S91F, D95A | D86N |
| SQ-24-12 | S91F, D95A | S87R |
| SQ-24-13 | S91F, D95A | D86N |
| SQ-24-14 | S91F, D95A | S87R |
| SQ-24-17 | S91F, D95A | S87R |
| SQ-24-18 | S91F, D95A | D86N |
| SQ-24-19 | S91F, A92P, D95Y | S87I |
| SQ-24-21 | S91F, D95A | S87R |
| SQ-24-22 | S91F, D95A | S87R |
| SQ-24-23 | S91F, D95A | S87R |
| SQ-24-24 | S91F, D95A | S87R |
| SQ-24-27 | S91F, D95G | S87R |
| SW-23-01 | S91F, D95G | E91G |
| SW-23-02 | S91F, D95G | E91G |
| SW-23-04 | S91F, D95A | D86N |
| SW-23-05 | S91F, D95A | S87R |
| SW-23-06 | S91F, D95A | S87R |
| SW-23-07 | S91F, D95A | D86N |
| SW-23-08 | S91F, D95A | D86N |
| SW-23-09 | S91F, D95A | D86N |
| SW-23-10 | S91F, D95A | D86N |
| SW-23-11 | S91F, D95A | S87N |
| SW-23-12 | S91F, D95A | D86N |
| SW-23-13 | S91F, D95A | D86N |
| SW-23-16 | S91F, D95A | D86N |
| SW-23-17 | S91F, D95A | D86N |
| SW-23-18 | S91F, D95A | D86N |
| SW-23-19 | S91F, D95G | D86N |
| SW-23-22 | S91F, D95A | S87R |
| SW-23-24 | S91F, D95A | D86N |
| SW-23-28 | S91F, D95A | D86N |
| SW-23-29 | S91F, D95G | E91G |
| SW-23-30 | S91F, D95A | E91Q, S87N |
| SW-24-02 | S91F, D95A | S87N, E91G |
| SW-24-04 | S91F, D95A | D86N |
| SW-24-05 | S91F, D95G | S87R |
| SW-24-06 | S91F, D95A | D86N |
| SW-24-07 | S91F, D95G | E91G |
| SW-24-08 | S91F, D95A | S87R |
| SW-24-10 | S91F, D95A | D86N |
| SW-24-11 | S91F, D95A | S87R |
| SW-24-12 | S91F, D95A | S87R |
| SW-24-13 | S91F, D95A | D86N |
| SW-24-14 | S91F, D95G | E91G |
| SW-24-15 | S91F, D95A | S87R |
| SW-24-16 | S91F, D95A | D86N |
| SW-24-17 | S91F, A92P, D95Y | S87N |
| SW-24-19 | S91F, A92P, D95Y | S87N |
| SW-24-20 | S91F, D95A | D86N |
| SW-24-23 | S91F, D95A | S87R |
| SW-24-24 | S91F, D95A | D86N |
| SW-24-25 | S91F, D95A | D86N |
| SY-23-24 | S91F, D95A | S87R |
| SY-24-02 | S91F, D95A | D86N |
| SY-24-04 | S91F, D95N | S87R |
| SY-24-05 | S91F, D95A | D86N |
| SY-24-06 | S91F, D95A | G85C, S87R |
| SY-24-07 | S91F, D95A | D86N |
| SY-24-10 | S91F, D95A | S87R |
| SY-24-11 | S91F, D95G | E91G |
| SY-24-13 | S91F, D95N | S87R |
| SY-24-17 | S91F, A92P, D95Y | S87R |
| SY-24-18 | S91F, A92P, D95A | S87R |
| SYN-22-01 | S91F, D95A | S87R |
| SYN-22-02 | S91F, D95A | D86N |
| SYN-22-03 | S91F, D95G | S87R |
| SYN-22-04 | S91F, D95A | D86N |
| SYN-22-05 | S91F, D95A | S87R |
| SYN-22-07 | S91F, D95A | D86N |
| SYN-22-08 | S91F, D95A | S87R |
| SYN-22-10 | S91F, D95A | S87R |
| SYN-22-13 | S91F, D95A | S87R |
| SYN-22-14 | S91F, D95G | S87R |
| SYN-22-15 | S91F, A92P, D95Y | S87N |
| SYN-22-16 | S91F, A92P, D95Y | S87N |
| SYN-23-01 | S91F, D95A | D86N |
| SYN-23-02 | S91F, D95A | S87R |
| SYN-23-04 | S91F, A92P, D95Y | S87N |
| SYN-24-01 | S91F, A92P, D95Y | S87N |
| SYN-24-02 | S91F, A92P, D95Y | S87N |
| SYN-24-04 | S91F, D95G | G120R, S87R |
| SYN-24-05 | S91F, D95G | S87R |
| SZX-22-01 | S91F, D95A | S87R |
| SZX-22-02 | S91F, D95N | D86N |
| SZX-22-03 | S91F, D95A | D86N |
| SZX-22-05 | S91F, D95A | D86N |
| SZX-22-06 | S91F, D95A | D86N |
| SZX-22-08 | S91F, D95A | S87R |
| SZX-22-09 | S91F, D95A | S87R |
| SZX-22-10 | S91F, D95A | S87R |
| SZX-22-12 | S91F, D95A | S87I, E91G |
| SZX-22-13 | S91F, D95N | D86N |
| SZX-22-14 | S91F, D95G | E91G |
| SZX-22-15 | S91F, D95A | S87R |
| SZX-22-16 | S91F, A92P, D95Y | S87N |
| SZX-22-18 | S91F, D95A | D86N |
| SZX-22-19 | S91F, D95A | S87C |
| SZX-22-20 | S91F, D95A | S87R |
| SZX-22-21 | S91F, D95A | G85C |
| SZX-22-23 | S91F, D95A | D86N |
| SZX-22-24 | S91F, D95A | G85D, S87R |
| SZX-22-25 | S91F, D95A | D86N |
| SZX-22-26 | S91F, D95G | D86N |
| SZX-22-27 | S91F, A92P, D95Y | S87N |
| SZX-22-30 | S91F, D95A | S87R |
| SZX-23-05 | S91F, D95A | S87R |
| SZX-23-06 | S91F, D95A | D86N |
| SZX-23-07 | S91F, D95A | S87R |
| SZX-23-08 | S91F, D95A | S87R |
| SZX-23-10 | S91F, D95A | S87R |
| SZX-23-11 | S91F, D95A | D86N |
| SZX-23-12 | S91F, D95A | D86N |
| SZX-23-13 | S91F, D95A | D86N |
| SZX-23-14 | S91F, D95G | S87R |
| SZX-23-15 | S91F, D95N | S87R |
| SZX-23-16 | S91F, D95A | G85C, S87R |
| SZX-23-17 | S91F, D95A | S87R |
| SZX-23-19 | S91F, D95A | D86N |
| SZX-23-20 | S91F, D95A | D86N |
| SZX-23-21 | S91F, D95A | S87R |
| SZX-23-22 | S91F, A92P, D95Y | S87N |
| SZX-23-23 | S91F, D95A | S87R |
| SZX-23-24 | S91F, D95A | S87N |
| SZX-23-25 | S91F, D95G | S87R |
| SZX-23-26 | S91F, D95A | D86N |
| SZX-23-28 | S91F, D95A | S87N |
| SZX-23-29 | S91F, D95A | D86N |
| SZX-23-30 | S91F, D95A | S87C |
| SZX-24-01 | S91F, D95A | D86N |
| SZX-24-02 | S91F, A92P, D95Y | S87N |
| SZX-24-03 | S91F, D95A | S87R |
| SZX-24-04 | S91F, D95A | D86N |
| SZX-24-07 | S91F, D95A | D86N |
| SZX-24-08 | S91F, D95A | D86N |
| SZX-24-18 | S91F, D95A | D86N |
| SZX-24-19 | S91F, D95G | S87R |
| SZX-24-20 | S91F, D95A | D86N |
| SZX-24-21 | S91F, D95A | G85C, S87R |
| SZX-24-22 | S91F, A92P, D95A | G85C, S87R |
| SZX-24-23 | S91F, D95A | S87R |
| SZX-24-24 | S91F, D95A | S87R |
| SZX-24-26 | S91F, D95A | S87I, E91G |
| SZX-24-27 | S91F, A92P, D95Y | S87N |
| SZX-24-28 | S91F, A92P, D95Y | S87I |
| SZX-24-29 | S91F, D95A | E91G |
| SZX-24-30 | S91F, D95G | E91G |
| SZX-24-31 | S91F, A92P, D95Y | S87I |
| SZX-24-32 | S91F, A92P, D95Y | S87I |
| SZX-24-34 | S91F, D95A | S87I |
| SZX-24-36 | S91F, D95A | S87R |
| SZX-24-37 | S91F, A92P, D95Y | S87I |
| SZX-24-38 | S91F, A92P, D95Y | S87N |
| SZX-24-39 | S91F, D95G | D86N |
| SZX-24-41 | S91F, D95A | D86N |
| SZX-24-42 | S91F, D95A | S87R |
| SZX-24-43 | S91F, D95A | S87R |
| SZX-24-44 | S91F, D95A | S87R |
| SZX-24-45 | S91F, D95A | S87R |
| SZX-24-46 | S91F, D95A | D86N |
| SZX-24-47 | S91F, D95A | S87R |
| SZX-24-48 | S91F, A92P, D95Y | S87I |
| SZX-24-53 | S91F, D95A | S87R |
| SZX-24-55 | S91F, A92P, D95Y | S87R |
| SZX-24-59 | S91F, D95A | S87R |
| SZX-24-60 | S91F, D95A | S87N |
| SZX-24-61 | S91F, D95A | S87R |
| SZX-24-62 | S91F, D95A | G85D, S87R |
| SZX-24-63 | S91F, D95G | S87R |
| SZX-24-66 | S91F, A92P, D95Y | S87I |
| SZX-24-69 | S91F, A92P, D95Y | S87I |
| SZX-24-70 | S91F, D95A | S87R |
| SZX-24-71 | S91F, D95A | S87R |
| SZX-24-73 | S91F, D95A | S87R |
| SZX-24-74 | S91F, D95A | S87R |
| TR-23-01 | S91F, D95A | D86N |
| TR-23-02 | S91F, D95A | S87R |
| TR-24-06 | S91F, D95A | D86N |
| TR-24-07 | S91F, D95G | E91G |
| TR-24-08 | S91F, D95A | D86N |
| TR-24-09 | S91F, D95A | D86N |
| TR-24-10 | S91F, D95A | S87R |
| TR-24-11 | S91F, D95A | S87R |
| TR-24-12 | S91F, D95A | S87R |
| TR-24-13 | S91F, A92P, D95Y | S87N |
| XDH-22-01 | S91F, D95A | D86N |
| XDH-22-03 | S91F, D95A | S87R |
| XDH-22-04 | S91F, D95A | S87R |
| XDH-22-05 | S91F, D95A | S87R |
| XDH-22-06 | S91F, D95A | S87N |
| XDH-22-07 | S91F, D95A | S87R |
| XDH-22-08 | S91F, D95A | S87R |
| XDH-22-09 | S91F, D95A | D86N |
| XDH-22-10 | S91F, D95A | D86N |
| XDH-24-04 | S91F, D95A | S87R |
| XDH-24-05 | S91F, D95G | D86N |
| XDH-24-06 | S91F, D95A | S87N |
| XDH-24-07 | S91F, D95A | S87R |
| XDH-24-08 | S91F, D95A | D86N |
| XDH-24-09 | S91F, D95A | S87R |
| XDH-24-10 | S91F, D95A | S87R |
| XDH-24-11 | S91F, D95N | S87R |
| XDH-24-12 | S91F, D95A | S87R |
| XH-23-17 | S91F, D95G | D86N |
| XH-23-18 | S91F, D95A | G85C |
| XH-23-19 | S91F, D95A | S87R |
| XH-23-20 | S91F, D95G | D86N |
| XH-23-22 | S91F, D95A | S87R |
| XH-24-13 | S91F, D95A | S87R |
| XH-24-16 | S91F, D95A | D86N |
| XH-24-17 | S91F, D95A | S87R |
| XH-24-20 | S91F, D95A | G85C |
| XH-24-21 | S91F, D95A | S87R |
| XH-24-22 | S91F, D95A | S87R |
| XH-24-24 | S91F, D95A | D86N |
| XH-24-25 | S91F, D95A | D86N |
| XZX-23-01 | S91F, D95G | E91G |
| XZX-23-02 | S91F, A92P, D95Y | S87N |
| XZX-23-04 | S91F, D95A | S87R |
| XZX-24-04 | S91F, D95A | S87R |
| ZJJ-23-02 | S91F, D95A | S87R |
| ZJJ-24-01 | S91F, D95A | S87R |
| ZP-23-06 | S91F, D95A | S87R |
| ZP-23-07 | S91F, A92P, D95Y | S87N |
| ZP-23-09 | S91F, A92P, D95Y | S87N |
| ZP-24-06 | S91F, D95A | D86N |
| ZP-24-07 | S91F, D95A | S87R |


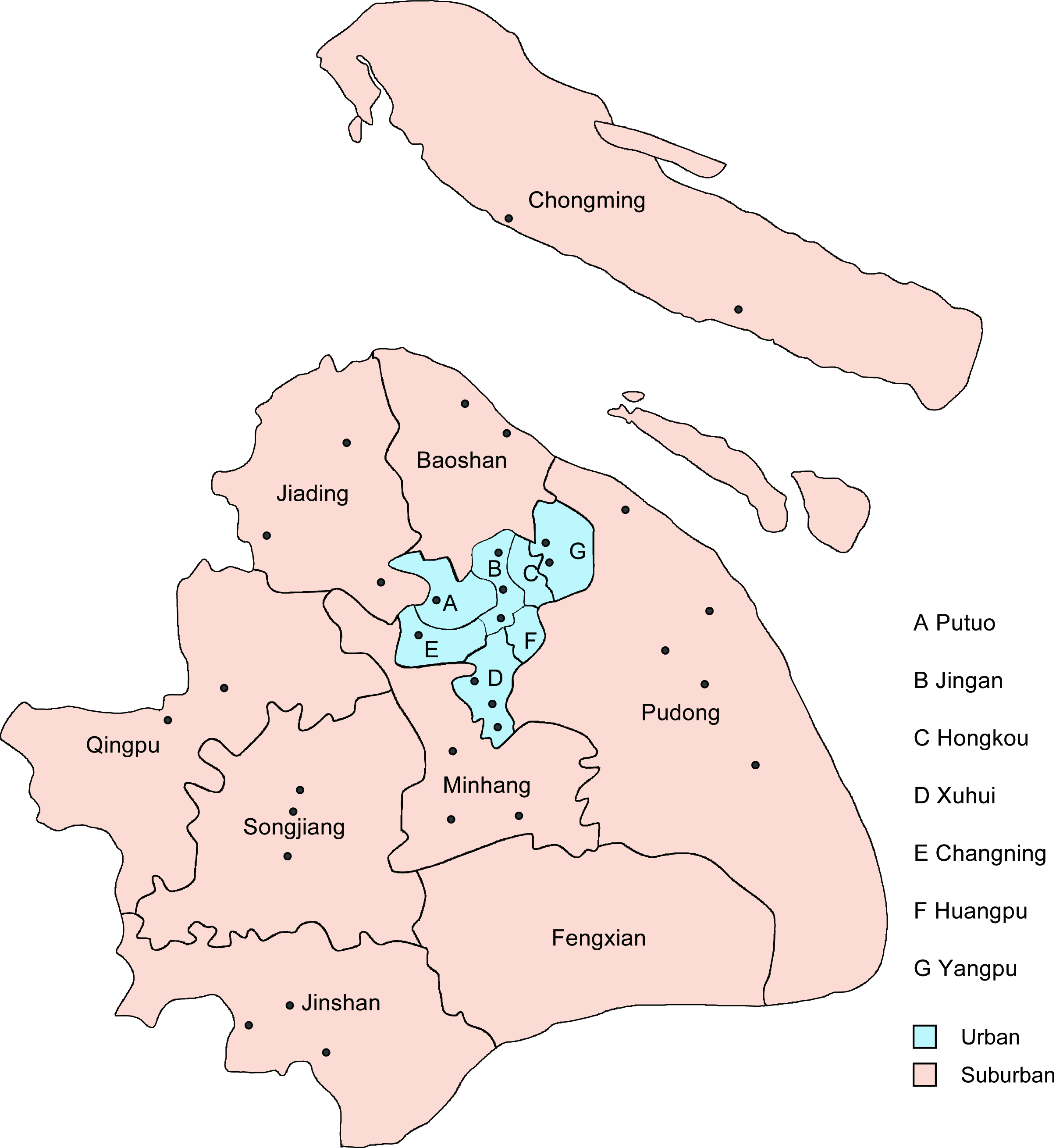


Figure S1: Geographical distribution of source hospitals for 989 *N. gonorrhoeae* isolates in Shanghai
